# Dietary patterns and physical activity in relation to renal and metabolic outcomes among older adults with type 2 diabetes and chronic kidney disease: a systematic review

**DOI:** 10.3389/fnut.2026.1858619

**Published:** 2026-09-16

**Authors:** Jinglong Li, Qisen Zhu, Jian Ding, Nik Nur Izzati Nik Mohd Fakhruddin

**Affiliations:** 1 Industrial Design College, LuXun Academy of Fine Arts, Shenyang, China; 2 Faculty of Education, Universiti Kebangsaan Malaysia, Bangi, Selangor, Malaysia; 3 Jeffrey Cheah School of Medicine & Health Sciences, Monash University, Subang Jaya, Malaysia

**Keywords:** chronic kidney disease, clinical nutrition, dietary patterns, older adults, physical activity, type 2 diabetes

## Abstract

**Introduction:**

Lifestyle modification is increasingly recognized as a critical component in the management of chronic kidney disease (CKD), particularly among elderly individuals with type 2 diabetes, a population at high risk of disease progression and comorbidities. While pharmacological treatment remains central, growing evidence highlights the role of dietary patterns and physical activity in potentially influencing renal and metabolic outcomes. However, the extent and consistency of these effects remain unclear, particularly in older diabetic populations. This systematic review aimed to synthesize evidence from both intervention and observational studies examining dietary patterns, dietary interventions, physical activity, and related lifestyle exposures associated with renal and metabolic outcomes in older adults with type 2 diabetes and chronic kidney disease.

**Methods:**

A comprehensive literature search was conducted across PubMed, EMBASE, Scopus, Web of Science, and the Cochrane Library. A total of 31 eligible studies were included in the final analysis.

**Results:**

Healthier dietary patterns—particularly Mediterranean, plant-based, low-sodium, and moderated protein approaches—were generally associated with favorable renal and metabolic outcomes, although the certainty of evidence varied across themes. Sodium restriction reduced blood pressure and albuminuria, while moderated protein intake was associated with favorable renal outcomes when nutritional status was carefully monitored. Physical activity interventions improved glycemic control, blood pressure, and functional capacity, with possible indirect benefits for renal function. Evidence regarding antioxidant supplementation and very low-carbohydrate diets remains limited and inconclusive.

**Conclusion:**

Dietary patterns and physical activity are associated with renal and metabolic outcomes among elderly individuals with type 2 diabetes. These findings suggest that nutrition-focused lifestyle interventions may be considered supportive components of routine care, although stronger evidence is required. However, further large-scale, high-quality, and long-term studies are needed to establish definitive recommendations and develop tailored intervention strategies for this high-risk population.

**Systematic Review Registration:**

https://www.crd.york.ac.uk/PROSPERO/view/CRD420251087535, identifier PROSPERO (CRD420251087535).

## Introduction

Chronic kidney disease (CKD) remains a pressing global health concern, affecting approximately 10% of the world’s population (1). It is typically diagnosed when a patient’s estimated glomerular filtration rate (eGFR) falls below 60 mL/min/1.73 m^2^ for at least 3 months (2). Type 2 diabetes mellitus is one of the leading causes of CKD, particularly among elderly populations, where age-related physiological decline compounds metabolic and vascular complications (3). The increasing prevalence of diabetes and aging populations has contributed significantly to the growing burden of CKD, with global mortality due to kidney disease rising by over 40% from 1990 to 2017 (4). CKD is projected to become the fifth leading cause of death worldwide by 2040, underscoring the urgent need for effective, accessible, and non-invasive strategies for disease management (5).

Conventional CKD management often includes pharmacological interventions, blood pressure control, and, in advanced stages, renal replacement therapy such as dialysis or transplantation (6). However, these options are often costly, invasive, and associated with reduced quality of life, especially in elderly patients who may also suffer from frailty, cognitive decline, and polypharmacy. Moreover, CKD is frequently diagnosed at later stages when intervention options are limited (7). Therefore, early-stage interventions that focus on modifiable lifestyle factors have gained prominence in clinical practice and research.

Emerging evidence suggests that a healthy diet and regular physical activity are associated with favorable renal and metabolic outcomes among older adults with type 2 diabetes and CKD, improving glycemic control and reducing cardiovascular risk among type 2 diabetic elderly patients (8). Dietary patterns such as the Mediterranean, DASH, and plant-based diets have been linked to better renal outcomes (9), while moderate-intensity exercise has shown benefits in reducing albuminuria, improving blood pressure, and enhancing metabolic function (10). Despite these findings, studies have reported varied outcomes due to differences in intervention types, durations, and adherence levels. Furthermore, many existing studies lack focus on elderly populations with both diabetes and CKD, leading to questions about generalizability and effectiveness.

This review synthesizes evidence from both intervention and observational studies to examine how dietary patterns, nutritional strategies, and physical activity are associated with renal and metabolic outcomes in older adults with type 2 diabetes and chronic kidney disease. Because studies exclusively targeting older adults with type 2 diabetes and CKD remain limited, this review also considers clinically relevant indirect evidence from broader CKD or type 2 diabetes populations when the investigated lifestyle exposures and renal outcomes are directly relevant to the target population. Such evidence is interpreted separately according to its degree of applicability.

## Methodology

### Protocol registration

The protocol for this review was registered in the PROSPERO database (registered ID: CRD420251246555) and conducted in strict accordance with the Preferred Reporting Items for Systematic Reviews and Meta-Analyses (PRISMA) (11).

### Review question and PIO framework

The review question and eligibility criteria were developed using the Population–Intervention/Exposure–Outcome (PIO) framework. Unlike conventional intervention-focused systematic reviews that typically adopt the PIO framework, the present review synthesizes evidence from both intervention studies (e.g., randomized controlled trials and non-randomized intervention studies) and observational studies (e.g., cohort and cross-sectional studies) (12). The included literature evaluates not only the effectiveness of dietary and physical activity interventions but also the associations between lifestyle exposures, including dietary patterns, physical activity, sedentary behavior, and renal or metabolic outcomes.

Because many of the eligible observational studies did not include a comparison group, a comparator (C) was not universally applicable across all included study designs. Consequently, the PIO framework was considered more appropriate than the traditional PICO framework for formulating the review question, developing the search strategy, and defining the eligibility criteria while maintaining methodological consistency across mixed evidence.

Accordingly, the review question was formulated as follows: Among older adults with type 2 diabetes and chronic kidney disease (population), how are dietary interventions, dietary patterns, physical activity interventions, and lifestyle-related exposures (Intervention/Exposure) associated with renal and metabolic outcomes (outcomes)?

The PIO framework applied in this review comprised the following components, as listed in Table 1:

**Table 1:** PIO components.

PIO components	Definition	Key search concepts and terms
Population	Older adults with type 2 diabetes and chronic kidney disease.	Older adults; elderly; aged; senior; geriatric; Type 2 diabetes; T2DM
Intervention/ exposure	Diet and lifestyle-related factors, including dietary practices, nutrition, a healthy diet, physical activity, exercise, and lifestyle interventions.	Diet; nutrition; healthy diet; dietary patterns; physical activity; exercise; lifestyle
Outcomes	Kidney-related outcomes, including chronic kidney disease, diabetic kidney disease, kidney disease, and other renal health outcomes associated with diet, nutrition, physical activity, or lifestyle factors.	Chronic kidney disease; CKD; diabetic kidney disease; DKD; kidney disease; renal/kidney outcomes

Population (P): Older adults with type 2 diabetes and chronic kidney disease.

Intervention/Exposure (I): Diet and lifestyle-related factors, including dietary practices, nutrition, healthy diet, physical activity, exercise, and lifestyle interventions.

Outcomes (O): Kidney-related outcomes, including chronic kidney disease, diabetic kidney disease, kidney disease, and other renal health outcomes associated with diet, nutrition, physical activity, or lifestyle factors.

Eligible study designs, including randomized controlled trials, non-randomized intervention studies, prospective cohort studies, and cross-sectional studies, were specified separately in the eligibility criteria rather than within the PIO framework. The complete database-specific search strings as originally executed are presented in Table 2 and Supplementary Material 2.

**Table 2:** Database-specific search string as executed during the systematic search (2015–2025).

Database	Search string as executed	Results
PubMed	(“older adult* with diabete*” OR “senior* with diabete*” OR “geriatric* with diabete*” OR “diabete* old*” OR “diabete* elder*” OR “diabete* aged” OR “diabete* senior*” OR “elderly with diabetes” OR “elderly patient* with type 2 diabete*”) AND (diet* OR nutrition* OR “health diet*” OR activity OR exercise* OR lifestyle*) AND (“kidney* solution*” OR “chronic kidney*” OR kidney* OR “chronic kidney disease” OR CKD OR “diabetic kidney disease” OR DKD OR “kidney disease”) Filters applied: Full text, Address, Biography, Books and Documents, Case Reports, Clinical Study, Comparative Study, Interview, Introductory Journal Article, English, Humans, Aged: 65 + years, Exclude preprints.	918 results
EMBASE	(‘older adult* with diabete*’ OR ‘senior* with diabete*’ OR ‘geriatric* with diabete*’ OR ‘diabete* old*’ OR ‘diabete* elder*’ OR ‘diabete* aged’ OR ‘diabete* senior*’ OR ‘elderly with diabetes’ OR ‘elderly patient* with type 2 diabete*’) AND (diet* OR nutrition* OR ‘health diet*’ OR ‘activity’/exp. OR activity OR exercise* OR lifestyle*) AND (‘kidney* solution*’ OR ‘chronic kidney*’ OR kidney* OR ‘chronic kidney disease’/exp. OR ‘chronic kidney disease’ OR ckd OR ‘diabetic kidney disease’/exp. OR ‘diabetic kidney disease’ OR dkd OR ‘kidney disease’/exp. OR ‘kidney disease’) AND [2015–2025]/py AND (‘article’/it OR ‘article in press’/it) AND english:la AND [humans]/lim AND [clinical study]/lim AND ([controlled clinical trial]/lim OR [randomized controlled trial]/lim)	11 results
Scopus	TITLE-ABS-KEY ((“older adult* with diabete*” OR “senior* with diabete*” OR “geriatric* with diabete*” OR “diabete* old*” OR “diabete* elder*” OR “diabete* aged” OR “diabete* senior*” OR “elderly with diabetes” OR “elderly patient* with type 2 diabete*”) AND (diet* OR nutrition* OR “health diet*” OR activity OR exercise* OR lifestyle*) AND (“kidney* solution*” OR “chronic kidney*” OR kidney* OR “chronic kidney disease” OR CKD OR “diabetic kidney disease” OR DKD OR “kidney disease”))	75 results
Web of Science	((ALL = (“older adult* with diabete*” OR “senior* with diabete*” OR “geriatric* with diabete*” OR “diabete* old*” OR “diabete* elder*” OR “diabete* aged” OR “diabete* senior*” OR “elderly with diabetes” OR “elderly patient* with type 2 diabete*”)) AND ALL = (diet* OR nutrition* OR “health diet*” OR activity OR exercise* OR lifestyle*)) AND ALL = (“kidney* solution*” OR “chronic kidney*” OR kidney* OR “chronic kidney disease” OR CKD OR “diabetic kidney disease” OR DKD OR “kidney disease”)	70 results
Cochrane	(older NEXT adult* NEXT with NEXT diabete* OR senior* NEXT with NEXT diabete* OR geriatric* NEXT with NEXT diabete* OR diabete* NEXT old* OR diabete* NEXT elder* OR diabete* NEXT aged OR diabete* NEXT senior* OR elderly NEXT with NEXT diabetes OR elderly NEXT patient* NEXT with NEXT type 2 NEXT diabete*) AND (diet* OR nutrition* OR health NEXT diet* OR activity OR exercise* OR lifestyle*) AND (kidney* NEXT solution* OR chronic NEXT kidney* OR kidney* OR chronic NEXT kidney NEXT disease OR CKD OR diabetic NEXT kidney NEXT disease OR DKD OR kidney NEXT disease)	19 results

Search strings are reported as originally executed to ensure transparency and reproducibility of the review process. Database-specific syntax and operators varied across platforms. Some search expressions were formulated during the original iterative search process and are therefore retained verbatim rather than retrospectively modified, as modifying the executed strategies would no longer correspond to the reported retrieval counts.

### Data sources and search strategy

This systematic review was conducted in accordance with the Preferred Reporting Items for Systematic Reviews and Meta-Analyses (PRISMA 2020) Statement (12). The complete PRISMA Checklist is provided in Supplementary Material 1. A comprehensive literature search was independently conducted by two reviewers in PubMed, EMBASE, Scopus, Web of Science Core Collection, and the Cochrane Library. The final search was completed on 31 December 2025, covering studies published between 1 January 2015 and 31 December 2025. Only English-language publications involving human participants were included, as reliable translation of non-English studies was beyond the scope of the review.

The search strategy was developed through an iterative process to achieve a balance between comprehensiveness and specificity. The initial search terms were refined following discussions with a healthy diet and nutrition specialist to ensure that the terminology accurately reflected the clinical and research context of dietary management and kidney health among older adults with diabetes. The final search strings incorporated multiple synonymous terms for the target population (e.g., older adults, elderly, seniors, and geriatric populations), lifestyle-related exposures (e.g., diet, nutrition, exercise, physical activity, and lifestyle), and kidney-related conditions (e.g., chronic kidney disease, diabetic kidney disease, CKD, DKD, and other kidney-related terms). This iterative refinement was undertaken to maximize the retrieval of relevant studies while minimizing irrelevant records. The search strategy was designed to capture a broad range of potentially relevant literature while avoiding an overly restrictive search that could exclude relevant evidence. This iterative approach was intended to balance the sensitivity and specificity of the literature search while maintaining transparency in the reporting of the search process. The 10-year search window was selected to capture contemporary evidence on the influence of healthy dietary patterns and physical activity on CKD management among older adults with type 2 diabetes. As a search result listed in Table 2, a total of 1,093 articles were found. To improve transparency and reproducibility, the complete database-specific search strategies are reported exactly as executed in Supplementary Material 2, including the Boolean combinations, database-specific syntax, applied filters, search dates, and retrieval results. Because the searches had already been completed and formed the basis of the study selection process, the original executed search expressions were retained rather than retrospectively reformulated, which would have produced retrieval results inconsistent with the reported PRISMA flow. Database-specific syntax, controlled vocabulary terms (e.g., MeSH and Emtree), Boolean operators, truncation, and filters were adapted according to each database platform. The complete search strategies are provided in Supplementary Material 2. This approach preserved consistency between the reported search strategies and the original retrieval and screening records, although it may have resulted in the omission of studies that could have been identified using a revised search strategy.

This review was conducted as a mixed evidence synthesis, incorporating both intervention and observational studies to provide a comprehensive understanding of the relationships between diet, nutrition, physical activity, lifestyle factors, and kidney-related outcomes among older adults with diabetes. Intervention studies were included to evaluate the effectiveness of diet-, nutrition-, physical activity-, exercise-, and lifestyle-related interventions, whereas observational studies were included to examine associations between these lifestyle-related factors and kidney-related outcomes, including chronic kidney disease (CKD), diabetic kidney disease (DKD), and other renal health outcomes. Given the methodological diversity of the included studies, findings were synthesized narratively, with intervention and observational evidence interpreted according to their respective study designs rather than being combined quantitatively.

### Eligibility criteria

Following the revision of the PIO eligibility criteria, all included studies were re-screened against the updated criteria. Studies providing indirect but clinically relevant evidence were retained and explicitly interpreted as indirect evidence during the narrative synthesis. In the process of selecting the articles, 1,093 articles were screened and excluded according to the criteria listed in Table 3. Studies involving broader CKD or type 2 diabetes populations were retained when they provided indirect evidence relevant to lifestyle management in older adults with diabetic CKD. These studies were interpreted cautiously during evidence synthesis. Before proceeding to the next screening process, duplicate articles were removed. For those articles that went through the screening process, the full texts of each study were screened and evaluated by the first and second authors to ensure the accuracy and consistency of the selection process.

**Table 3:** Inclusion and exclusion criteria.

Inclusion criteria
Studies involving older adults with diabetes, including elderly, senior, geriatric, or aged populations. Studies involving chronic kidney disease (CKD), diabetic kidney disease (DKD), or other kidney-related conditions. Studies investigating diet, nutrition, healthy diet, physical activity, exercise, or lifestyle interventions or exposures. Studies examining the relationship between diet-, nutrition-, physical activity-, or lifestyle-related factors and kidney health or renal outcomes.

Exclusion criteria
Studies involving CKD populations without relevant diabetes or older-adult subgroup information. Studies that did not investigate diet, nutrition, healthy diet, physical activity, exercise, or lifestyle factors. Full-text articles unavailable. Grey literature, including theses, dissertations, unpublished reports, conference proceedings, and abstracts. Publications not written in English. Secondary literature, including systematic reviews, narrative reviews, scoping reviews, meta-analyses, editorials, opinion articles, commentaries, and perspective papers.

### Study selection

A total of 1,093 publications were retrieved at the preliminary stage of article screening. Two reviewers independently screened all retrieved records following predefined inclusion and exclusion standards, with duplicate entries eliminated prior to formal screening. After removing redundant records, the two authors independently assessed the full text of each remaining study to ensure reliable and consistent literature selection outcomes.

### Assessment of study quality

Considering the methodological diversity of the included studies, study quality was appraised using the Mixed Methods Appraisal Tool (MMAT), 2018 version (13). The MMAT is specifically designed for systematic reviews that include multiple research designs and enables the assessment of qualitative, quantitative, and mixed-methods studies within a single framework. The included studies comprised randomized controlled trials, quantitative non-randomized studies, and quantitative descriptive studies. Accordingly, only the corresponding MMAT domains were applied.

The methodological appraisal focused on the appropriateness of the study design, participant selection, data collection methods, outcome measurement, risk of bias, and the credibility of the reported findings. Two reviewers independently assessed the methodological quality of all eligible studies, with any discrepancies resolved through discussion until consensus was achieved, thereby enhancing the consistency and reliability of the appraisal process (14).

The results of the quality assessment were not used to exclude studies from the review. Instead, they were considered during the interpretation and synthesis of the findings to provide appropriate context for evaluating the strength of the available evidence.

### Assessment of overall certainty of evidence

To further evaluate the overall strength of the evidence, the Grading of Recommendations Assessment, Development, and Evaluation (GRADE) framework was applied (15). The GRADE approach assesses the certainty of evidence by considering several domains, including study design, risk of bias, consistency of findings, indirectness of evidence, imprecision, and other factors that may influence confidence in the results. Based on these domains, the certainty of evidence is classified into four levels: high, moderate, low, or very low.

Given the substantial heterogeneity among the included studies in terms of study design, participant characteristics, intervention or exposure types, and reported outcomes, the GRADE assessment was interpreted with caution. Accordingly, the resulting certainty ratings should be regarded as an overall indication of the strength of the available evidence rather than definitive conclusions regarding the effectiveness or association of the investigated interventions and exposures.

## Results

### Screening the article results

A total of 1,093 records were retrieved and exported to Microsoft Excel for duplicate removal. After removing 318 duplicates, 775 records remained for title screening. Six hundred thirty-five unrelated articles were removed based on the title screen. The remaining 140 articles were screened through abstract review; 67 articles were removed in this process. The remaining 73 articles were thoroughly assessed by the two independent reviewers, and only those articles meeting the criteria were included. In cases of contrasting opinions, other reviewers were consulted to resolve any disparity in the results through voting. However, any unresolved conflicts were ultimately addressed through consensus, ensuring that all decisions were mutually agreed upon by the research team. Secondary screening was performed by reading the full text of the articles thoroughly. Furthermore, back screening was conducted by checking the references for potential articles. Finally, 31 selected studies were included in the systematic review. The final selected studies are provided in Supplementary Material 3. The detailed PRISMA flow chart is available in Figure 1.

**Figure 1:**
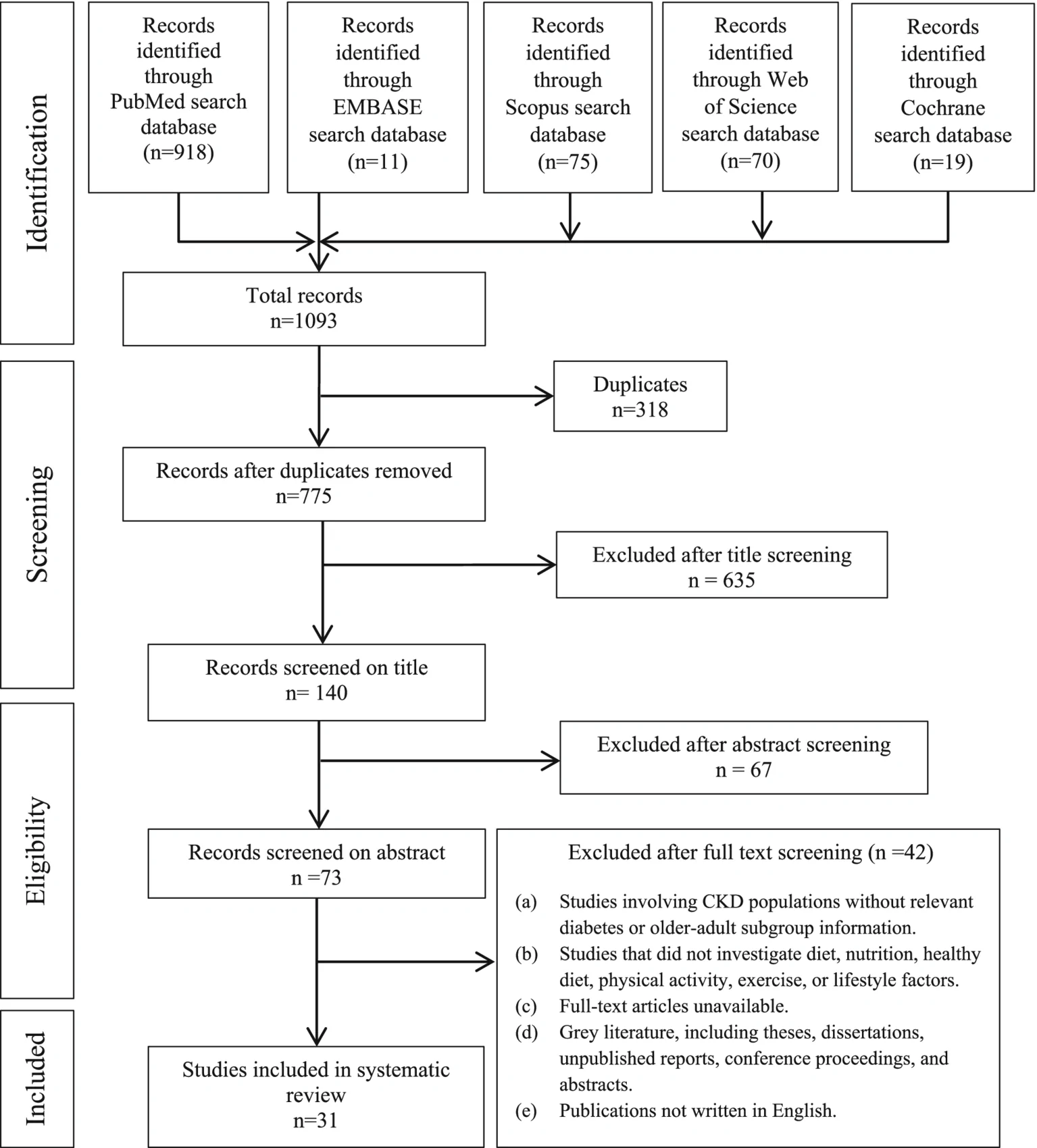
Flow chart of search strategy based on PRISMA flow diagram.

The methodological quality of the 31 included studies was assessed using the Mixed Methods Appraisal Tool (MMAT) 2018, and the results are summarized in Table 4. The appraisal evaluated the appropriateness of the study design, sampling strategy, outcome measurement, and management of confounding factors according to the relevant MMAT criteria.

**Table 4:** Mixed methods appraisal tools (MMAT) scoring ratings.

MMAT checklist evaluation criteria (“Y” = Yes, “N” = No, and “U” = Cannot tell)																		
	1. Screening questions		2. Quantitative randomized controlled trials (RCTs)					3. Quantitative non-randomized					4. Quantitative descriptive					Score (%)
Authors (Year)	S1	S2	1.1	1.2	1.3	1.4	1.5	3.1	3.2	3.3	3.4	3.5	4.1	4.2	4.3	4.4	4.5	
Quantitative randomized controlled trials (RCTs)																		
Podadera-Herreros et al. (16)	Y	Y	Y	Y	Y	U	Y											80%
Meng et al. (19)	Y	Y	Y	Y	U	Y	Y											80%
Tauchi et al. (22)	Y	Y	Y	U	Y	Y	Y											80%
Bellizzi et al. (26)	Y	Y	Y	U	U	Y	Y											60%
Zainordin et al. (29)	Y	Y	U	Y	Y	Y	Y											80%
Nataraj et al. (39)	Y	Y	U	U	Y	Y	Y											60%
Miraghajani et al. (36)	Y	Y	Y	Y	Y	Y	U											80%
Khatami et al. (33)	Y	Y	U	Y	U	Y	Y											60%
Quantitative non-randomized studies																		
Ding et al. (17)	Y	Y						Y	N	Y	Y	Y						80%
Rouhani et al. (21)	Y	Y						Y	Y	Y	U	Y						80%
Piccoli et al. (25)	Y	Y						N	Y	Y	Y	Y						80%
Tay et al. (32)	Y	Y						Y	Y	U	Y	Y						80%
Zainordin et al. (30)	Y	Y						Y	Y	Y	U	U						60%
Zainordin et al. (31)	Y	Y						Y	Y	U	Y	Y						80%
Saran et al. (27)	Y	Y						Y	Y	U	Y	Y						80%
Kimura et al. (40)	Y	Y						Y	Y	Y	U	Y						80%
Palakodeti et al. (44)	Y	Y						Y	Y	Y	Y	N						80%
Aydemir et al. (41)	Y	Y						Y	Y	Y	U	Y						80%
Anderton et al. (43)	Y	Y						Y	Y	Y	Y	N						80%
Rouhani et al. (37)	Y	Y						Y	Y	Y	U	Y						80%
Shah et al. (46)	Y	Y						Y	N	Y	Y	Y						80%
Quantitative descriptive studies																		
Cao et al. (18)	Y	Y											Y	U	Y	Y	Y	80%
Wilkinson et al. (20)	Y	Y											Y	Y	U	Y	Y	80%
Malhotra et al. (23)	Y	Y											Y	U	Y	Y	Y	80%
Møller et al. (24)	Y	Y											Y	Y	Y	U	U	60%
Kanauchi et al. (28)	Y	Y											Y	Y	U	U	Y	60%
Leehey et al. (38)	Y	Y											Y	Y	U	U	Y	60%
Jiménez-Osorio et al. (35)	Y	Y											Y	Y	U	U	Y	60%
Bao et al. (34)	Y	Y											Y	Y	Y	U	N	60%
Beetham et al. (42)	Y	Y											Y	Y	U	U	Y	60%
Wong et al. (45)	Y	Y											Y	N	Y	U	Y	60%

The MMAT findings are presented using three response categories: “Yes” (Y), “No” (N), and “Cannot tell” (U). Overall, the included studies demonstrated acceptable methodological quality, with most meeting the majority of the MMAT criteria applicable to their respective study designs. Common strengths included appropriate research designs, robust data collection procedures, valid outcome measurements, and, where relevant, satisfactory integration of qualitative and quantitative findings in mixed-methods studies.

Nevertheless, several methodological limitations were identified. These mainly related to limited participant representativeness, insufficient reporting of strategies for addressing potential confounding factors, and inadequate methodological detail, particularly in pilot and feasibility studies with relatively small sample sizes. Among the mixed-methods studies, some also provided limited information regarding the integration and interpretation of qualitative and quantitative findings, resulting in uncertainty about the coherence of the combined evidence.

Although methodological limitations were identified, none were considered sufficiently serious to justify study exclusion. All 31 studies met the predefined eligibility criteria and contributed evidence relevant to the objectives of this review. Consequently, the MMAT assessment was used to inform the interpretation of the findings, allowing the results to be considered in light of the methodological strengths and limitations of the included evidence while identifying areas for improvement in future research.

### Data extraction

Data were systematically extracted from all included studies (research articles, randomized controlled trials, cohort studies, and intervention studies) using a standardized data extraction form developed in Microsoft Excel. Two reviewers independently extracted and cross-checked study information to ensure accuracy and consistency.

Extracted baseline characteristics included participants’ mean age, gender distribution, body mass index (BMI), duration of type 2 diabetes, stage of chronic kidney disease (CKD), presence of comorbidities, and lifestyle-related factors where reported.

Study characteristics collected comprised author(s), year of publication, brief summary, objectives, implications, methodology, outcomes, and limitations. Outcome measures extracted included renal function indicators (eGFR, serum creatinine), metabolic parameters (HbA1c, blood pressure, body weight), and additional health-related outcomes such as quality of life, physical functioning, fatigue/energy levels, and inflammatory markers (e.g., C-reactive protein). Where reported, both baseline and post-intervention values were extracted to enable comparison of changes across studies.

### Narrative synthesis approach

Following the Synthesis Without Meta-analysis (SWiM) reporting guideline, findings were synthesized using a structured narrative approach. Studies were grouped according to intervention/exposure type, study design, and outcome domain. For each outcome, the direction of effect (beneficial, harmful, or no clear association), reported outcome measures, follow-up duration, and methodological limitations were extracted and compared. Quantitative pooling was not undertaken because of substantial heterogeneity in study populations, intervention characteristics, exposure definitions, outcome measurements, and study designs.

### Study selection and characteristics

Initial comprehensive database searches yielded a total of 1,093 articles. Following a rigorous screening process, 31 full-text articles were retrieved for detailed assessment against the predefined inclusion and exclusion criteria. The included studies comprised research articles, randomized controlled trials (RCTs), prospective cohort studies, cross-sectional analyses, and secondary analyses of large databases. Sample sizes ranged from small pilot trials (*n* < 50) to large population-based cohorts (>10,000 participants). Most studies included older adults with type 2 diabetes and varying stages of chronic kidney disease (CKD), with several specifically focusing on elderly populations or reporting subgroup analyses for older adults. The interventions investigated across these studies encompassed a range of approaches, including dedicated healthy diet programs, structured physical activity protocols, and integrated combined approaches. All interventions were specifically designed to improve chronic kidney disease (CKD) outcomes in elderly individuals diagnosed with type 2 diabetes.

Renal outcomes were primarily measured using estimated glomerular filtration rate (eGFR; creatinine- or cystatin-C–based), albuminuria/proteinuria, CKD stage progression, or incidence of end-stage renal disease (ESRD). Secondary outcomes frequently included glycemic control (HbA1c), blood pressure, lipid profiles, inflammatory markers, oxidative stress biomarkers, and anthropometric measures. The findings were synthesized into four major thematic domains: (1) dietary patterns and diet quality; (2) macronutrient strategies (protein, sodium, carbohydrate modification); (3) anti-inflammatory and antioxidant nutrition approaches; and (4) physical activity and behavioral self-management in CKD management.

The included studies comprised both intervention and observational designs. Intervention studies evaluated structured dietary and physical activity programs designed to improve renal or metabolic outcomes, whereas observational studies examined associations between habitual dietary patterns, physical activity, sedentary behavior, or nutritional exposures and CKD-related outcomes. Accordingly, findings were synthesized according to evidence type and thematic similarity rather than pooled quantitatively.

### Overall certainty of evidence (GRADE assessment)

As described in the previous section, four thematic categories were identified. To evaluate the overall certainty of the evidence across the included studies, the Grading of Recommendations Assessment, Development and Evaluation (GRADE) framework was applied. The results of the GRADE assessment are presented in Table 5 in accordance with established GRADE guidelines (15). Overall, the certainty of the evidence ranged from moderate to very low across the four thematic categories. The dietary patterns and diet quality theme was rated as having moderate-certainty evidence because it included relatively consistent findings from one long-term randomized controlled trial along with several well-conducted observational studies. In contrast, the remaining three themes were rated as low- or very low-certainty evidence because of methodological heterogeneity, the predominance of observational studies and small intervention trials, variations in intervention and exposure characteristics, and imprecision in the reported renal outcome measures.

**Table 5:** Certainty of evidence across thematic categories assessed using the GRADE framework.

Outcome/thematic category	Number of studies	Risk of bias	Inconsistency	Indirectness	Imprecision	Certainty
Dietary patterns and diet quality	6	Not serious	Not serious	Not serious	Serious	⊕⊕⊕◯ Moderate
Macronutrient strategies (protein, sodium, carbohydrate modification)	11	Serious	Serious	Not serious	Serious	⊕⊕◯◯ Low
Anti-inflammatory and antioxidant nutrition approaches	5	Serious	Serious	Not serious	Serious	⊕⊕◯◯ Low
Physical activity and behavioral self-management in CKD management	9	Serious	Serious	Serious	Serious	⊕◯◯◯ Very low

Evidence was downgraded primarily due to small sample sizes, exploratory study designs, limited use of randomized controlled trials, and heterogeneity in intervention types and outcome measures.

### Theme 1: Dietary patterns and overall dietary quality in relation to CKD outcomes

Across the six included studies listed in Table 6, stronger adherence to overall “healthy” dietary patterns (e.g., Mediterranean-style patterns or higher diet-quality indices) was consistently linked with better kidney-related markers and/or lower CKD risk among diabetic or CKD-affected populations, supporting the idea that whole-diet quality matters more than single nutrients.

**Table 6:** Studies that focused on the dietary patterns with CKD.

Author(s)/year	Thematic focus	Objectives	Design/sample	Research methods	Key findings/outcomes	Limitation
Cao et al. (18)	Diet and CKD progression	Evaluate dietary pattern impact on renal outcomes in T2DM elderly	Cohort; elderly with T2DM and CKD	Dietary assessment; eGFR monitoring	Slower CKD progression with healthy diet	Observational design
Ding et al. (17)	Diet pattern and renal function	Examine health diet effects on CKD indicators	Intervention; T2DM with CKD	Renal biomarkers	Improved eGFR and BP control	Small sample size
Meng et al. (19)	Diet pattern intervention	Assess diet + exercise on metabolic and renal outcomes	RCT; elderly T2DM	Lifestyle modification; lab tests	Better glycemic and BP control	Short follow-up
Wilkinson et al. (20)	Diet pattern, food intake and CKD	Investigate dietary patterns and kidney health	Prospective study; CKD adults	Diet quality scoring; renal function tests	Diet adherence linked to better renal outcomes	Self-reported diet bias
Rouhani et al. (21)	Three Diet Quality and diabetes	Evaluate health diet lifestyle modification in T2DM	Clinical intervention; diabetic adults	Nutrition counseling + PA	Improved HbA1c and BP	Limited elderly subgroup analysis
Podadera-Herreros et al. (16)	Mediterranean diet as strategy	Assess dietary pattern and effectiveness	Pilot trial; elderly CKD	Functional tests	Improved body function and BP	Pilot scale; limited generalizability

Evidence from a long-term randomized dietary trial suggests that a Mediterranean dietary pattern can meaningfully preserve renal function in high-risk cardiometabolic patients. In a secondary analysis of the CORDIOPREV randomized controlled trial, participants with both T2DM and obesity experienced a smaller decline in eGFR and a reduction in uACR under a Mediterranean diet compared with a low-fat diet, indicating potential benefits for slowing renal deterioration in metabolically complicated subgroups (16).

Observational findings in diabetic populations further reinforce these protective associations. In a Chinese cross-sectional study of individuals with diabetes, dietary pattern analysis showed that those with higher adherence to a plant-based dietary pattern had lower odds of renal impairment, using low eGFR and albuminuria indicators (17). In an elderly-specific cohort (≥65 years) with T2DM, a large community-based study identified distinct patterns: a “balanced” pattern was associated with lower CKD prevalence, whereas an “imbalanced” pattern (low dietary diversity) was associated with higher CKD prevalence and higher odds of reduced eGFR (18). Similarly, Cao et al. reported that a balanced dietary pattern characterized by higher consumption of fruits, dairy products, eggs, fish, and shellfish was associated with significantly lower odds of CKD, whereas an imbalanced dietary pattern was linked to increased CKD prevalence among elderly Chinese patients with type 2 diabetes (19).

Population-level analyses using national datasets also showed that “diet quality scoring” is clinically relevant for renal outcomes. A comprehensive NHANES-based analysis comparing multiple dietary pattern indices (2000–2020) reported that higher-quality diet patterns were consistently associated with more favorable CKD-related outcomes, supporting the robustness of diet quality constructs across different scoring systems (19). Complementing this, a CKD-stage–focused cluster analysis in England demonstrated that food intake patterns vary across CKD stages, emphasizing that dietary pattern differentiation is linked with disease severity and may be useful for stage-sensitive dietary guidance (20).

Finally, evidence from CKD clinic populations suggests that “diet quality indices” based on broader intake patterns can relate to CKD severity. A cross-sectional Iranian study comparing three CKD-relevant diet quality indices reported that higher animal protein intake per body weight showed a marginal association with higher CKD stage, illustrating how diet quality constructs can reflect clinically meaningful differences in renal status (21).

Overall, intervention studies demonstrated beneficial effects of healthier dietary patterns on renal outcomes, whereas observational studies consistently reported associations between higher diet quality and better kidney health. Collectively, these complementary findings support a potential association between overall dietary quality and more favorable renal outcomes, although causal inference remains limited, particularly for the observational evidence.

### Theme 2: Nutritional strategies for CKD in elderly patients with type 2 diabetes

Across the 11 included studies presented in Table 7, nutritional strategies for diabetic CKD management mainly clustered around (i) protein quantity/quality and protein restriction approaches, (ii) sodium restriction, and (iii) carbohydrate restriction (very low-carbohydrate diets). Overall, the evidence supports individualized dietary prescriptions that balance potential renal benefits with the high risk of malnutrition in older adults.

**Table 7:** Studies that focused on the nutritional strategies for CKD.

Author(s)/year	Thematic focus	Objectives	Design/sample	Research methods	Key findings/outcomes	Limitation
Tauchi et al. (22)	Dietary intervention and CKD	Examine nutritional strategies’ impact on renal function; supports dietary modification in CKD management	RCT; T2DM elderly	Controlled diet; renal biomarkers (eGFR, creatinine)	Improved renal parameters	Short duration
Malhotra et al. (23)	Higher protein intake and glycemic control	Assess nutritional strategies’ effects on metabolic and renal outcomes	Intervention; CKD patients	HbA1c and BP measurement	Improved glycemic and BP control	Small sample
Møller et al. (24)	Lower protein intake and CKD	Investigate adherence to nutritional strategies and CKD progression	Cohort study; CKD adults	Diet score analysis; longitudinal follow-up	Slower CKD progression	Self-reported diet bias
Piccoli et al. (25)	Lower protein intake and CKD	Evaluate nutritional strategies + exercise synergy in T2DM with CKD	RCT; elderly diabetics	Lifestyle program; lab assessments	Improved eGFR and insulin sensitivity	Limited follow-up
Bellizzi et al. (26)	Protein intake and kidney outcomes	Assess protein restriction effects on renal decline	Clinical trial; CKD patients	Controlled protein diet; renal markers	Reduced renal deterioration	Adherence issues
Tay et al. (32)	Low Carbohydrate Compared With a High Carbohydrate Diet and CKD	Examine impact of nutritional strategies on kidney function	Intervention study	Supervised aerobic sessions; BP and eGFR	Improved cardiovascular and renal indicators	Non-randomized design
Zainordin et al. (29)	Very-low-carbohydrate diet and CKD	Test plant-based dietary pattern on metabolic risk	RCT; overweight adults	Dietary intervention; insulin resistance test	Improved insulin sensitivity	Non-CKD population
Zainordin et al. (30)	Very-low-carbohydrate diet and renal outcomes	Determine effect of nutrition counseling on CKD management	Prospective study	Counseling + monitoring	Better dietary adherence and BP control	Observational bias
Zainordin et al. (31)	Very-low-carbohydrate diet and CKD	Evaluate nutritional strategies in elderly CKD	Pilot trial; elderly CKD	Strength program; functional tests	Improved muscle strength and function	Pilot scale
Saran et al. (27)	Dietary sodium restriction and CKD	Investigate sodium intake and blood pressure control in CKD	Clinical study; CKD adults	Sodium-controlled diet; BP tracking	Improved BP regulation	Short-term study
Kanauchi et al. (28)	Progression of renal dysfunction and the importance of salt restriction and CKD	Analyze association between nutritional strategies and CKD progression	Cohort study	Questionnaires; renal follow-up	Higher activity linked to slower decline	Self-report measurement

Protein intake and protein restriction—“renal protection vs. malnutrition risk”—is the first section to discuss. Evidence on dietary protein intake was mixed, but most studies suggest that protein moderation may be beneficial for kidney outcomes in advanced diabetic nephropathy, provided that nutritional status is protected. In a cohort of patients with advanced diabetic nephropathy, lower dietary protein intake was associated with a reduced risk of initiating renal replacement therapy; however, it was linked to higher mortality among patients who were already malnourished, indicating that protein restriction may be harmful if nutritional reserves are low (22). This directly highlights a key clinical tension in elderly diabetic CKD: Restricting protein too aggressively can increase vulnerability to protein-energy wasting.

Two additional observational analyses support cautious protein moderation in high-risk groups. In a large nested case–control study, higher protein intake was associated with increased incident ESRD risk among Black adults with diabetes, suggesting that higher habitual protein exposure may accelerate progression in vulnerable diabetic populations (23). However, not all older groups showed harm: In a 1-year intervention substudy of prediabetic older adults, higher protein intake increased urea-related markers but was not associated with declines in creatinine clearance, eGFR, ACR, or serum creatinine, suggesting that moderate increases in protein may not automatically translate into kidney function loss in earlier-risk older adults (24). Together, these studies indicate that baseline renal risk and nutritional status likely modify the impact of protein intake.

Second, the low-protein diets and ketoacid supplementation—feasible and potentially protective—are an important aspect. Evidence from clinical and practice-based studies suggests that low-protein diets (LPD) can be feasible in diabetic CKD and may be associated with favorable renal outcomes when carefully implemented. An observational comparison of diabetic and non-diabetic CKD patients following a moderately restricted LPD reported that outcomes were broadly comparable when adherence was good, supporting the practical feasibility of LPD use in diabetes rather than treating diabetes as a contraindication (25). More specifically, a low-protein diet supplemented with ketoacids (LPD-KA) in CKD stages 3–5 demonstrated improvements in urea and phosphate with stable albumin and no adverse nutritional signal, and fasting glucose decreased without increased insulin requirements in diabetic participants—suggesting that LPD-KA can be a structured approach to protein moderation while maintaining nutritional status (26). Overall, these findings support individualized protein management in elderly diabetic CKD, emphasizing careful monitoring for malnutrition and functional decline.

Third, regarding sodium restriction—consistent benefits for BP control and renal risk markers. Compared with protein, sodium restriction showed more consistent benefits across study designs. A randomized crossover trial in stage 3–4 CKD found that a sodium-restricted diet reduced 24-h systolic blood pressure by 10.8 mmHg compared with the usual diet (95% CI, −17.0 to −4.6; *p* = 0.001), supporting sodium restriction as a clinically meaningful strategy in moderate-to-advanced CKD (27). In diabetic kidney disease specifically, a retrospective analysis showed that salt intake and urinary protein excretion were independently associated with annual creatinine clearance decline, and that intensive dietary education reduced salt intake differences and improved decline trajectories among “decliners,” highlighting the practical importance of sustained salt reduction and education-based adherence support (28). Collectively, these studies support sodium restriction as a core dietary component in diabetic CKD management, with potential downstream benefits for BP and proteinuria-related progression risk.

Finally, very low-carbohydrate diets show short-term metabolic gains, with renal outcomes mostly stable but long-term uncertainty. Evidence from short-term trials indicates that very low-carbohydrate approaches can yield rapid metabolic improvements in diabetic kidney disease, but long-term renal outcomes remain uncertain—especially in older CKD populations. In a 12-week randomized controlled trial in DKD, the very-low-carbohydrate diet produced a median HbA1c reduction of 1.3 percentage points (IQR 1.1), compared with 0.7 percentage points (IQR 1.25) in the control group, while body weight decreased by 4.0 kg (IQR 3.9) versus 0.2 kg (IQR 4.2; *p* < 0.001), respectively. Renal outcomes, including eGFR, remained stable, with no statistically significant deterioration during the intervention (29). A related renal-outcome–focused report from the same 12-week RCT similarly found no deterioration in eGFR, urine protein indices, or blood pressure, alongside improved glycemic control and weight outcomes—supporting short-term safety in mild-to-moderate DKD (30). A companion analysis emphasized short-term metabolic efficacy (weight, waist, visceral adiposity, inflammation), although lipid changes (e.g., LDL increases) warrant monitoring in clinical application (31).

Longer-term evidence is still limited and somewhat indirect for established DKD. In obese adults with type 2 diabetes without overt kidney disease, a 12-month trial comparing a very low-carbohydrate/high-protein diet with a higher-carbohydrate diet found no adverse differences in creatinine, eGFR, or albumin excretion, suggesting renal marker stability over 1 year in non-CKD T2DM populations (32). Overall, the carbohydrate restriction literature supports short-term metabolic benefits with generally stable short-term renal indices, but the evidence regarding sustained renal outcomes and long-term safety in elderly DKD remains inconclusive and requires longer follow-up trials.

In a word, theme 2 supports an individualized nutritional strategy in elderly diabetic CKD: protein moderation may be considered as part of an individualized nutritional strategy, but its potential renal benefits must be balanced against the risk of malnutrition; sodium restriction appears consistently beneficial for BP and progression-related factors; and very low-carbohydrate diets show short-term metabolic improvements with stable renal markers, but long-term renal outcomes remain underdefined in elderly DKD.

### Theme 3: Anti-inflammatory and antioxidant nutritional supplementation in CKD

As listed in Table 8, five studies examined nutritional approaches that specifically target oxidative stress and inflammation, two central mechanisms implicated in the development and progression of diabetic nephropathy and proteinuric CKD.

**Table 8:** Studies that focused on anti-inflammatory and antioxidant nutrition approaches to CKD.

Author(s)/year	Thematic focus	Objectives	Design/sample	Research methods	Key findings/outcomes	Limitation
Miraghajani et al. (36)	Dietary pattern and CKD	Evaluate effect of nutritional approaches on renal and metabolic outcomes	Intervention study; CKD/T2DM adults	Supervised program; renal and metabolic markers	Improved glycemic control and physical function	Small sample; short duration
Jiménez-Osorio et al. (35)	Dietary pattern and kidney health	Assess impact of nutritional approaches adherence on CKD progression	Observational cohort; CKD patients	Diet assessment tools; eGFR monitoring	Healthier diet associated with slower renal decline	Self-reported dietary data
Khatami et al. (33)	Combined lifestyle intervention	Examine nutritional approaches + exercise effects on metabolic and renal parameters	Clinical trial; diabetic adults	Lifestyle modification program; lab measurements	Improved HbA1c, BP, and renal indicators	Limited follow-up
Rouhani et al. (37)	Lifestyle counseling in CKD	Determine effect of nutritional lifestyle education on CKD management	Prospective intervention; CKD patients	Nutrition and PA counseling; clinical follow-up	Better dietary adherence and BP control	Non-randomized design
Bao et al. (34)	Anti-inflammatory and antioxidant nutrition	Examine the association between nutritional and DKD in elderly patients with diabetes	Cross-sectional; 960 elderly (≥65 years) patients with diabetes (NHANES)	NHANES data; serum lycopene measured by HPLC; multivariable logistic regression	Moderate serum cis-lycopene was associated with lower odds of DKD, while total and trans-lycopene showed no significant association	Cross-sectional design; cannot infer causality; residual confounding possible

High-dose antioxidant therapy showed biomarker-level improvements in diabetic nephropathy. In a randomized, double-blind, placebo-controlled trial, high-dose vitamin E (1,200 IU/day) administered for 12 weeks improved oxidative stress– and inflammation-related biomarkers in patients with diabetic nephropathy, alongside modest stabilization signals in kidney-injury–related markers (33). Overall, these findings suggest that vitamin E may reduce oxidative burden in DN, although longer follow-up is required to determine whether these biomarker improvements translate into meaningful and sustained renal outcomes.

Additional evidence supporting the potential role of antioxidant nutrition was provided by a cross-sectional NHANES study of elderly patients (≥65 years) with diabetes. Bao et al. reported that moderate serum cis-lycopene concentrations were significantly associated with lower odds of diabetic kidney disease (DKD), whereas total and trans-lycopene showed no significant associations. These findings suggest that dietary antioxidants such as lycopene may be associated with favorable renal outcomes through potential anti-inflammatory and antioxidative mechanisms, although prospective studies are needed to establish causality (34).

Evidence for phytochemical anti-inflammatory supplementation was also supportive but preliminary. In an 8-week randomized, double-blind pilot trial involving non-diabetic and diabetic proteinuric CKD, curcumin supplementation (320 mg/day) improved redox-related outcomes and was linked to the activation of antioxidant defense pathways (including Nrf2-related mechanisms), even though conventional renal endpoints such as proteinuria and eGFR showed limited short-term change (35). This pattern suggests curcumin may exert early mechanistic benefits (redox regulation) that might precede measurable renal outcome changes, but the clinical significance remains uncertain without larger and longer trials.

A nutrition-based microbiome strategy also demonstrated promising changes in renal and metabolic markers in diabetic nephropathy. In a randomized controlled clinical trial among patients with type 2 diabetic nephropathy, consumption of probiotic soy milk (fortified with *Lactobacillus plantarum*) for 8 weeks was associated with improvements in selected renal function biomarkers and metabolic control indicators compared with control soy milk (36). Although short-term and biomarker-focused, this study supports the potential of probiotic-enriched functional foods as an adjunct strategy for DKD management.

Finally, one study approached inflammation from the “whole-diet inflammatory potential” perspective rather than single supplements. A cross-sectional analysis found that a higher Dietary Inflammatory Index (DII)—reflecting a more pro-inflammatory overall diet—was associated with poorer renal function and indicators consistent with CKD progression risk (37). This supports the broader interpretation that anti-inflammatory dietary exposures (not only supplements) may be relevant for CKD trajectory.

Across these five studies, antioxidant and anti-inflammatory nutritional strategies—including dietary supplementation, functional foods, antioxidant biomarkers, and diet-related inflammatory burden—were consistently associated with improvements in oxidative stress and inflammatory markers, with supportive evidence for favorable renal outcomes. However, the overall evidence remains limited by the predominance of short-term interventions, observational study designs, relatively small sample sizes, and the limited number of studies conducted specifically among older adults with diabetes and CKD.

### Theme 4: Physical activity and behavioral self-management in CKD management

Table 9 shows the nine included studies. Exercise-based interventions and behavioral self-management factors were consistently linked to improved metabolic control, physical function, and—more variably—renal outcomes in CKD populations, including those with diabetes. Overall, the evidence suggests that physical activity is generally feasible in CKD and is associated with improved cardiometabolic profiles, although evidence for direct renal benefits remains variable.

**Table 9:** Studies that focused on the physical activity and behavioral self-management in CKD.

Author(s)/year	Thematic focus	Objectives	Design/sample	Research methods	Key findings/outcomes	Limitation
Kimura et al. (40)	Exercise and CKD progression	Examine impact of structured physical activity on renal decline; supports exercise in CKD care	Prospective intervention; CKD adults	Supervised exercise; eGFR and BP monitoring	Slower renal decline; improved BP	Short-term follow-up
Beetham et al. (42)	Physical activity and mortality risk	Assess association between activity level and CKD outcomes	Cohort study; CKD patients	PA assessment; longitudinal follow-up	Higher activity linked to lower mortality	Observational bias
Palakodeti et al. (44)	Lifestyle modification in T2DM	Evaluate diet + exercise in metabolic control	Clinical intervention; diabetic adults	Lifestyle counseling; HbA1c and BP tests	Improved glycemic and BP control	Small sample
Nataraj et al. (39)	Combined lifestyle intervention	Determine effect of structured program on renal and metabolic markers	RCT; elderly T2DM with CKD	Diet + PA intervention; lab tests	Improved eGFR and insulin sensitivity	Limited duration
Aydemir et al. (41)	Sodium restriction and BP	Investigate sodium reduction in CKD management	Clinical trial; CKD adults	Sodium-controlled diet; BP tracking	Improved BP regulation	Short-term design
Anderton et al. (43)	Exercise therapy in CKD	Assess safety and effectiveness of exercise training	Pilot trial; CKD patients	Aerobic training; functional tests	Improved physical capacity	Small pilot study
Leehey et al. (38)	Dietary protein management	Examine protein intake and kidney function	Observational study; CKD adults	Dietary intake analysis; renal markers	Lower protein linked to slower decline	Self-reported diet
Shah et al. (46)	Lifestyle education	Evaluate impact of education on CKD self-management	Prospective study; CKD patients	Nutrition and PA education; follow-up	Improved adherence and BP control	Non-randomized
Wong et al. (45)	Physical activity and renal outcomes	Analyze relationship between exercise and CKD progression	Cohort study	Activity questionnaires; renal follow-up	Active patients had better outcomes	Measurement bias

First, regarding the exercise interventions—metabolic and functional benefits, with emerging renal signals. Randomized and interventional studies showed that structured exercise programs improved key cardiometabolic outcomes relevant to CKD progression, including glycemic control, blood pressure, body composition, and functional capacity. In obese diabetic CKD patients, structured exercise led to clinically meaningful improvements in cardiometabolic risk profiles and physical performance, supporting exercise as a feasible component of CKD management in high-risk diabetic populations (38). Similarly, exercise-based rehabilitation in type 2 diabetic nephropathy improved functional capacity and was associated with favorable trends in renal indicators over the intervention period, suggesting potential benefits for slowing functional decline in diabetic CKD (39).

Beyond diabetic nephropathy–focused trials, a multi-center randomized controlled trial evaluating exercise therapy stratified by renal pathology provided additional evidence that exercise interventions can be implemented safely in CKD populations, with renal effects that may differ by underlying kidney disease subtype (40). Complementing this, a trial examining combined lifestyle components (diet and exercise) in moderate-to-severe CKD found changes in metabolic and inflammatory-related biomarkers (e.g., adipocytokines), reinforcing the idea that lifestyle interventions may influence CKD outcomes through systemic pathways linked to inflammation and metabolic regulation (41).

Second, interpreting renal outcomes—measurement considerations (creatinine vs. cystatin-C). A key methodological issue is that exercise—especially when it increases or preserves muscle mass—may affect serum creatinine and complicate the interpretation of creatinine-based eGFR changes. One study explicitly compared creatinine- and cystatin-C–based eGFR estimates before and after a 12-month exercise intervention in CKD and highlighted that agreement between these measures can vary, underscoring the need for cautious interpretation when renal outcomes are evaluated in exercise trials (42). This is particularly relevant when reporting “renal improvements” because shifts in eGFR may partly reflect biomarker behavior rather than true changes in filtration.

Third, sedentary behavior and real-world activity patterns in diabetic CKD and dialysis. Observational evidence showed that sedentary behavior is highly prevalent among individuals with diabetic CKD and those receiving maintenance hemodialysis, reinforcing the need for targeted physical activity promotion and sedentary time reduction strategies—especially for older patients who may face additional barriers to sustained activity (43). In broader diabetes cohorts, longitudinal data also suggested that many patients remain inactive over time, and only a subset transitions to meeting recommended activity levels—highlighting implementation challenges and the need for supportive systems that help patients maintain activity behavior changes (44).

Finally, behavioral self-management: health literacy, adherence, and progression heterogeneity. Lifestyle effectiveness depends not only on intervention design but also on behavioral and contextual factors that influence adherence. Evidence linking health literacy with self-care behaviors suggests that patients with stronger literacy and self-management capacity demonstrate better engagement with recommended behaviors, which likely supports sustained lifestyle adherence in CKD management (45). Additionally, prospective observational work examining CKD progression trajectories found substantial heterogeneity in disease course, consistent with the idea that comorbidities, engagement patterns, and behavioral factors contribute to divergent outcomes over time (46).

Overall synthesis: Taken together, the evidence indicates that exercise interventions in CKD—including diabetic CKD—consistently improve metabolic control and physical function, with promising but not uniform renal signals. The interpretation of renal changes should consider the eGFR measurement method (creatinine vs. cystatin-C). Finally, sedentary behavior is common in diabetic CKD/dialysis, and long-term lifestyle effectiveness is strongly influenced by health literacy and self-management capacity, emphasizing the importance of behavioral support alongside exercise prescriptions (38–46).

### Summary of evidence direction and outcome effects

To provide a structured overview of the synthesized evidence, findings from the included studies were summarized according to thematic category, study design, outcome domains, direction of effect, and major evidence limitations. Following SWiM principles, this narrative synthesis approach was used because quantitative meta-analysis was not appropriate due to substantial heterogeneity in study populations, interventions/exposures, outcome measurements, and study designs. The summary highlights the overall pattern of evidence while distinguishing between intervention effects and observational associations (Table 10).

**Table 10:** Summary of evidence direction and outcome effects.

Theme	Study design	Outcomes	Representative quantitative findings	Direction of effect	Evidence limitations
1. Dietary patterns and diet quality	RCTs, cohort studies, cross-sectional studies	eGFR, albuminuria, CKD progression, HbA1c, blood pressure, metabolic indicators	High adherence to a balanced dietary pattern was associated with lower odds of CKD (OR = 0.73, 95% CI: 0.62–0.86; *p* < 0.001), reduced eGFR <60 mL/min/1.73 m^2^ (OR = 0.59, 95% CI: 0.46–0.76; *p* < 0.001), and albuminuria (OR = 0.77, 95% CI: 0.65–0.91; *p* = 0.002).	Generally beneficial association. Healthier dietary patterns (e.g., Mediterranean-style, plant-based, higher diet quality) were associated with slower renal decline, improved metabolic control, and reduced CKD risk indicators.	Moderate heterogeneity in dietary assessment methods; predominance of observational evidence; potential residual confounding; limited elderly diabetic CKD-specific studies.
2. Macronutrient strategies (protein, sodium, carbohydrate modification)	RCTs, clinical interventions, cohort studies	eGFR, creatinine, renal function markers, proteinuria, blood pressure, glycemic outcomes	Sodium restriction reduced 24-h systolic BP by 10.8 mmHg (95% CI: −17.0 to −4.6; *p* = 0.001). In a 12-week very-low-carbohydrate diet trial, median HbA1c decreased by 1.3 percentage points (IQR 1.1) versus 0.7 percentage points (IQR 1.25) in controls, while body weight decreased by 4.0 kg (IQR 3.9) versus 0.2 kg (IQR 4.2); renal indices, including eGFR, remained stable.	Mostly favorable but inconsistent effects. Protein modification, sodium restriction, and carbohydrate-controlled approaches showed potential benefits for renal function and metabolic control, although findings varied across interventions.	Small sample sizes; short intervention durations; variation in dietary protocols and adherence; limited long-term renal outcomes.
3. Anti-inflammatory and antioxidant nutritional supplementation	RCTs, pilot trials, cross-sectional studies	Oxidative stress biomarkers, inflammatory markers, renal biomarkers, DKD indicators	A higher pro-inflammatory dietary score was associated with greater odds of being in a higher CKD stage (OR = 2.12, 95% CI: 1.05–4.26; p for trend = 0.03). Curcumin (320 mg/day for 8 weeks) significantly attenuated lipid peroxidation in non-diabetic proteinuric CKD and enhanced antioxidant capacity in diabetic proteinuric CKD (*p* < 0.05), although eGFR and proteinuria did not significantly improve.	Potential beneficial effects. Antioxidant supplementation and anti-inflammatory nutritional approaches were associated with improved oxidative stress profiles and some supportive renal biomarker changes.	Evidence remains preliminary; mainly small-scale and short-term trials; biomarker-focused outcomes; limited studies specifically involving elderly adults with diabetic CKD.
4. Physical Activity and Behavioral Self-Management in CKD Management	RCTs, prospective interventions, cohort studies, observational studies	eGFR decline, blood pressure, physical function, glycemic control, quality of life, CKD progression indicators	In a 12-week exercise-based rehabilitation RCT, significant group × time effects were observed for 6-min walk distance (*F* = 619, *p* < 0.001), eGFR (*F* = 105.2, *p* < 0.001), serum creatinine (*F* = 174.8, *p* < 0.001), and serum urea (*F* = 261.4, *p* < 0.001), indicating improvements in functional capacity and renal function.	Generally favorable associations with improved physical function, metabolic control, and blood pressure regulation; evidence regarding CKD progression remains uncertain.	Considerable variation in exercise intensity, intervention duration, and behavioral components; limited blinding; observational studies susceptible to measurement and selection bias.

Overall, the evidence suggested generally favorable associations between lifestyle-related interventions or exposures and renal and metabolic outcomes; however, the strength and certainty of evidence varied across themes. Findings should be interpreted cautiously due to methodological limitations, including small sample sizes, short follow-up periods, heterogeneity in intervention characteristics, and the limited availability of studies specifically targeting older adults with type 2 diabetes and CKD.

## Discussion

This systematic review synthesized evidence from both intervention and observational studies. Intervention studies provided evidence regarding the effects of dietary and physical activity interventions, whereas observational studies contributed evidence on associations between lifestyle-related exposures and renal outcomes. Consequently, findings from observational studies should be interpreted as associative rather than causal, and the overall conclusions reflect the complementary nature of these two evidence types. Overall, the findings indicated generally favorable renal and metabolic outcomes associated with healthy dietary practices and physical activity, including improvements in eGFR, systolic blood pressure, glycemic control, and body weight in some studies. However, the magnitude, consistency, and certainty of these findings varied across study designs and outcomes. Because relatively few studies exclusively recruited older adults with both type 2 diabetes and CKD, the review also incorporated indirect evidence from broader CKD and type 2 diabetes populations. These studies were included to provide a more comprehensive understanding of lifestyle-related mechanisms but should be interpreted cautiously when generalizing findings to elderly diabetic CKD populations.

Our findings align with previous studies indicating that non-pharmacological interventions—particularly when sustained over time—can have clinically meaningful effects on kidney function and cardiovascular risk factors. Improvements in eGFR were observed in some studies, although these findings should be interpreted cautiously given differences in study design, measurement methods, and follow-up duration. Lower HbA1c levels also reflect improved insulin sensitivity and dietary compliance, which are essential in managing both diabetes and CKD.

The reduction in systolic BP observed in some studies indicates a potentially favorable cardiovascular outcome. However, whether these changes translate into sustained improvements in renal outcomes or slower CKD progression remains uncertain, given the limited follow-up and heterogeneous evidence base. Although improvements in diastolic BP and mental wellbeing were less consistent, this may be attributed to variations in intervention duration, intensity, or participant adherence across studies.

While quality of life improved in some studies, especially in the physical and energy domains, the impact on mental health was less conclusive. This highlights the need to integrate psychosocial support into lifestyle programs for elderly populations. The findings of this review should also be interpreted in light of the methodological quality and certainty of the available evidence. Although the MMAT assessment indicated that most included studies met acceptable methodological standards, variations in study design, intervention characteristics, and outcome measures contributed to overall heterogeneity. Similarly, the GRADE assessment indicated that the certainty of evidence ranged from moderate to very low across the four thematic categories. Although healthier dietary patterns, particularly Mediterranean-style diets, demonstrated consistent benefits for renal health, the certainty of this evidence was rated as moderate because much of it was derived from observational studies, supported by only a limited number of randomized controlled trials. The remaining thematic categories were supported by low- or very low-certainty evidence because of methodological heterogeneity, small intervention trials, and imprecision in reported renal outcomes. Therefore, the overall findings should be interpreted with appropriate caution, and additional well-designed randomized controlled trials are needed to strengthen the evidence base. Importantly, the findings should be interpreted in the context of substantial heterogeneity across the included evidence. Differences in study design, participant characteristics, intervention and exposure definitions, intervention duration, follow-up periods, and renal and metabolic outcome measures limited direct comparability across studies. In addition, the inclusion of evidence from randomized, non-randomized, and observational studies resulted in varying levels of methodological strength, while some evidence was derived from broader CKD or type 2 diabetes populations rather than exclusively from older adults with both conditions. Consequently, the certainty and generalizability of the findings vary across thematic categories, and conclusions based predominantly on observational or indirect evidence should be interpreted cautiously.

Despite these promising results, several limitations warrant discussion. First, substantial heterogeneity was observed across study designs, participant characteristics, intervention and exposure components, follow-up durations, and outcome measurements, limiting direct comparability and precluding quantitative pooling. This heterogeneity also reduced the certainty of the synthesized evidence and limits the extent to which the findings can be generalized across different elderly diabetic CKD populations. Second, some included studies were at high risk of bias, particularly in the reporting and attrition domains. Third, although the search strategy was developed through iterative refinement with input from a healthy diet and nutrition specialist to achieve an appropriate balance between sensitivity and specificity, the use of combined population-specific search terms may have excluded some relevant studies that described older adults, diabetes, or chronic kidney disease using different terminology. Therefore, despite the comprehensive search across multiple databases, the possibility of missing eligible studies cannot be entirely excluded. In addition, because the originally executed search strategies were retained rather than rerun using revised search terms, some potentially relevant studies that might have been identified through a revised search strategy may have been omitted. Lastly, the generalizability of findings may be limited due to the geographic concentration of trials in high-income countries.

## Practical implications

Despite these limitations, the findings have important implications for clinical practice and public health strategies.

Integrated Lifestyle Interventions: The results support the incorporation of combined healthy diet and physical activity programs into CKD management plans for type 2 diabetic elderly patients. Such interventions could be delivered through primary care, community centers, or telehealth platforms.

Personalized Nutrition and Exercise Plans: Tailoring interventions to individual patient needs, preferences, and physical limitations—particularly for older adults—may improve adherence and outcomes.

Multidisciplinary Collaboration: Dietitians, physiotherapists, nephrologists, and primary care physicians should collaborate to develop and monitor personalized lifestyle programs, ensuring safety and effectiveness for elderly populations with comorbidities.

Policy and Program Development: Health authorities may consider funding and supporting preventive lifestyle programs targeted at aging diabetic populations to reduce the burden of CKD progression and associated healthcare costs.

## Conclusion

This systematic review suggests that healthy dietary practices and structured physical activity may be associated with favorable renal and metabolic outcomes among older adults with type 2 diabetes and CKD. Potential benefits were observed across outcomes including eGFR, HbA1c, blood pressure, and physical function; however, the magnitude and certainty of these findings varied across study designs and thematic categories.

These findings suggest a potentially supportive role for non-pharmacological interventions in chronic disease management. However, given the heterogeneity of the included evidence and the moderate-to-very-low certainty across thematic categories, the findings should be interpreted cautiously. Future research should identify the most effective intervention components, evaluate their long-term sustainability, and determine their longer-term effects across more diverse populations and healthcare settings. Further well-designed and adequately powered studies are needed before lifestyle interventions can be more confidently integrated into standard CKD care pathways.

## Data Availability

The original contributions presented in the study are included in the article/Supplementary material, further inquiries can be directed to the corresponding author.
