# Supplementary material for: Dietary patterns and physical activity in relation to renal and metabolic outcomes among older adults with type 2 diabetes and chronic kidney disease: a systematic review
Source: Front Nutr. 2026 Sep 16;13:1858619. doi: 10.3389/fnut.2026.1858619 (PMC13623551; doi:10.3389/fnut.2026.1858619)
Supplement: Supplementary file 1 [file Table_1.docx]

| **Section and Topic** | **Item #** | **Checklist item** | **Location where item is reported** |
| --- | --- | --- | --- |
| **TITLE** | | |  |
| Title | 1 | Dietary Patterns and Physical Activity in Relation to Renal and Metabolic Outcomes among Older Adults with Type 2 Diabetes and Chronic Kidney Disease: A Systematic Review | title |
| **ABSTRACT** | | |  |
| Abstract | 2 | **Objective:** This systematic review aims to evaluate and synthesise current evidence on the effects of healthy dietary patterns and physical activity interventions in the management of chronic kidney disease (CKD) among elderly individuals with Type II diabetes.  **Design:** A systematic review was conducted in accordance with the Preferred Reporting Items for Systematic Reviews and Meta-Analyses (PRISMA) guidelines.  **Data sources:** PubMed, EMBASE, Scopus, Web of Science and the Cochrane Library were systematically searched for relevant studies from 1 January 2015 to 31 December 2025.  **Eligibility criteria:** Studies were included if they examined dietary interventions, nutritional strategies or structured physical activity programmes targeting elderly individuals with Type II diabetes and CKD, and reported renal or metabolic outcomes.  **Data extraction and synthesis:** Studies were assessed based on author, year, focus theme, research methodology, research design, outcomes, limitation. Owing to heterogeneity in study design and outcome reporting, a narrative synthesis was undertaken.  **Results:** Thirty studies met the inclusion criteria. Healthier dietary patterns—particularly Mediterranean, plant-based, low-sodium and moderated-protein approaches—were associated with improved renal and metabolic outcomes. Sodium restriction consistently reduced blood pressure and albuminuria, while moderated protein intake demonstrated potential renal protective effects with careful monitoring of nutritional status. Physical activity interventions were associated with improved glycaemic control, blood pressure regulation and functional capacity, with possible indirect benefits for renal function. Evidence supporting antioxidant supplementation and very low-carbohydrate diets remains limited and inconclusive.  **Conclusion:** This review demonstrates that healthy dietary patterns and physical activity interventions play a significant role in the management of CKD among elderly individuals with Type II diabetes. The findings support the integration of structured lifestyle-based strategies into routine clinical care to preserve renal function and improve metabolic outcomes. However, further large-scale, high-quality and long-term trials are required to establish definitive recommendations and develop tailored intervention models for this high-risk population. | abstract |
| **INTRODUCTION** | | |  |
| Rationale | 3 | Chronic Kidney Disease (CKD) remains a pressing global health concern, affecting approximately 10% of the world’s population. It is typically diagnosed when a patient's estimated glomerular filtration rate (eGFR) falls below 60 mL/min/1.73 m² for at least three months. Type II diabetes mellitus is one of the leading causes of CKD, particularly among elderly populations, where age-related physiological decline compounds metabolic and vascular complications. The increasing prevalence of diabetes and aging populations has contributed significantly to the growing burden of CKD, with global mortality due to kidney disease rising by over 40% from 1990 to 2017. CKD is projected to become the fifth leading cause of death worldwide by 2040, underscoring the urgent need for effective, accessible, and non-invasive strategies for disease management. | introduction |
| Objectives | 4 | This systematic review aims to investigate the effects of healthy diet and physical activity interventions on the management of chronic kidney disease among diabetic elderly. | abstract, objective. |
| **METHODS** | | |  |
| Eligibility criteria | 5 | Inclusion criteria:   (a) Studies involving older adults with diabetes, including elderly, senior, geriatric, or aged populations. (b) Studies involving chronic kidney disease (CKD), diabetic kidney disease (DKD), or other kidney-related conditions. (c) Studies investigating diet, nutrition, healthy diet, physical activity, exercise, or lifestyle interventions or exposures. (d) Studies examining the relationship between diet-, nutrition-, physical activity-, or lifestyle-related factors and kidney health or renal outcomes.   Exclusion Criteria   (e) Studies involving chronic kidney disease that did not focus on older adults with diabetes. (f) Studies that did not investigate diet, nutrition, healthy diet, physical activity, exercise, or lifestyle factors. (g) Full-text articles unavailable. (h) Grey literature, including theses, dissertations, unpublished reports, conference proceedings, and abstracts. (i) Publications not written in English.   Secondary literature, including systematic reviews, narrative reviews, scoping reviews, meta-analyses, editorials, opinion articles, commentaries, and perspective papers. | study selection |
| Information sources | 6 | PubMed, EMBASE, Scopus, web of science and Cochrane | table 1 |
| Search strategy | 7 | Present the full search strategies for all databases, registers and websites, including any filters and limits used.  Pubmed searching strings: ("older adult* with diabete*" OR "senior* with diabete*" OR "geriatric* with diabete*" OR "diabete* old*" OR "diabete* elder*" OR "diabete* aged" OR "diabete* senior*" OR "elderly with diabetes" OR "elderly patient* with type 2 diabete*") AND (diet* OR nutrition* OR "health diet*" OR activity OR exercise* OR lifestyle*) AND ("kidney* solution*" OR "chronic kidney*" OR kidney* OR "chronic kidney disease" OR CKD OR "diabetic kidney disease" OR DKD OR "kidney disease")  Filters applied: Full text, Address, Biography, Books and Documents, Case Reports, Clinical Study, Comparative Study, Interview, Introductory Journal Article, English, Humans, Aged: 65+ years, Exclude preprints.  EMBASE searching strings: ('older adult* with diabete*' OR 'senior* with diabete*' OR 'geriatric* with diabete*' OR 'diabete* old*' OR 'diabete* elder*' OR 'diabete* aged' OR 'diabete* senior*' OR 'elderly with diabetes' OR 'elderly patient* with type 2 diabete*') AND (diet* OR nutrition* OR 'health diet*' OR 'activity'/exp OR activity OR exercise* OR lifestyle*) AND ('kidney* solution*' OR 'chronic kidney*' OR kidney* OR 'chronic kidney disease'/exp OR 'chronic kidney disease' OR ckd OR 'diabetic kidney disease'/exp OR 'diabetic kidney disease' OR dkd OR 'kidney disease'/exp OR 'kidney disease') AND [2015-2025]/py AND ('article'/it OR 'article in press'/it) AND english:la AND [humans]/lim AND [clinical study]/lim AND ([controlled clinical trial]/lim OR [randomized controlled trial]/lim)  Scopus: TITLE-ABS-KEY ( ( "older adult* with diabete*" OR "senior* with diabete*" OR "geriatric* with diabete*" OR "diabete* old*" OR "diabete* elder*" OR "diabete* aged" OR "diabete* senior*" OR "elderly with diabetes" OR "elderly patient* with type 2 diabete*" ) AND ( diet* OR nutrition* OR "health diet*" OR activity OR exercise* OR lifestyle* ) AND ( "kidney* solution*" OR "chronic kidney*" OR kidney* OR "chronic kidney disease" OR CKD OR "diabetic kidney disease" OR DKD OR "kidney disease" ) )  Web of Science: ((ALL=("older adult* with diabete*" OR "senior* with diabete*" OR "geriatric* with diabete*" OR "diabete* old*" OR "diabete* elder*" OR "diabete* aged" OR "diabete* senior*" OR "elderly with diabetes" OR "elderly patient* with type 2 diabete*")) AND ALL=(diet* OR nutrition* OR "health diet*" OR activity OR exercise* OR lifestyle*)) AND ALL=("kidney* solution*" OR "chronic kidney*" OR kidney* OR "chronic kidney disease" OR CKD OR "diabetic kidney disease" OR DKD OR "kidney disease")  Cochrane: (older NEXT adult* NEXT with NEXT diabete* OR senior* NEXT with NEXT diabete* OR geriatric* NEXT with NEXT diabete* OR diabete* NEXT old* OR diabete* NEXT elder* OR diabete* NEXT aged OR diabete* NEXT senior* OR elderly NEXT with NEXT diabetes OR elderly NEXT patient* NEXT with NEXT type 2 NEXT diabete*) AND (diet* OR nutrition* OR health NEXT diet* OR activity OR exercise* OR lifestyle*) AND (kidney* NEXT solution* OR chronic NEXT kidney* OR kidney* OR chronic NEXT kidney NEXT disease OR CKD OR diabetic NEXT kidney NEXT disease OR DKD OR kidney NEXT disease) | methodology,  table 1 |
| Selection process | 8 | A total of 1093 publications were retrieved at the preliminary stage of article screening. Two reviewers independently screened all retrieved records following predefined inclusion and exclusion standards, with duplicate entries eliminated prior to formal screening. After removing redundant records, the two authors independently assessed the full text of each remaining study to guarantee reliable and consistent literature selection outcomes. | methodology |
| Data collection process | 9 | Two independent reviewers collected data from each report using a standardized extraction form. They worked separately to ensure accuracy, resolving discrepancies through discussion or with a third reviewer if needed. For missing or unclear information, reviewers contacted study investigators. No automation tools were used in the data collection process. | methodology |
| Data items | 10a | PubMed \| ("older adult* with diabete*" OR "senior* with diabete*" OR "geriatric* with diabete*" OR "diabete* old*" OR "diabete* elder*" OR "diabete* aged" OR "diabete* senior*" OR "elderly with diabetes" OR "elderly patient* with type 2 diabete*") AND (diet* OR nutrition* OR "health diet*" OR activity OR exercise* OR lifestyle*) AND ("kidney* solution*" OR "chronic kidney*" OR kidney* OR "chronic kidney disease" OR CKD OR "diabetic kidney disease" OR DKD OR "kidney disease") Filters applied: Full text, Address, Biography, Books and Documents, Case Reports, Clinical Study, Comparative Study, Interview, Introductory Journal Article, English, Humans, Aged: 65+ years, Exclude preprints. \| 918 results; EMBASE \| ('older adult* with diabete*' OR 'senior* with diabete*' OR 'geriatric* with diabete*' OR 'diabete* old*' OR 'diabete* elder*' OR 'diabete* aged' OR 'diabete* senior*' OR 'elderly with diabetes' OR 'elderly patient* with type 2 diabete*') AND (diet* OR nutrition* OR 'health diet*' OR 'activity'/exp OR activity OR exercise* OR lifestyle*) AND ('kidney* solution*' OR 'chronic kidney*' OR kidney* OR 'chronic kidney disease'/exp OR 'chronic kidney disease' OR ckd OR 'diabetic kidney disease'/exp OR 'diabetic kidney disease' OR dkd OR 'kidney disease'/exp OR 'kidney disease') AND [2015-2025]/py AND ('article'/it OR 'article in press'/it) AND english:la AND [humans]/lim AND [clinical study]/lim AND ([controlled clinical trial]/lim OR [randomized controlled trial]/lim) \| 11 results; Scopus \| TITLE-ABS-KEY ( ( "older adult* with diabete*" OR "senior* with diabete*" OR "geriatric* with diabete*" OR "diabete* old*" OR "diabete* elder*" OR "diabete* aged" OR "diabete* senior*" OR "elderly with diabetes" OR "elderly patient* with type 2 diabete*" ) AND ( diet* OR nutrition* OR "health diet*" OR activity OR exercise* OR lifestyle* ) AND ( "kidney* solution*" OR "chronic kidney*" OR kidney* OR "chronic kidney disease" OR CKD OR "diabetic kidney disease" OR DKD OR "kidney disease" ) ) \| 75 results; Web of Science \| ((ALL=("older adult* with diabete*" OR "senior* with diabete*" OR "geriatric* with diabete*" OR "diabete* old*" OR "diabete* elder*" OR "diabete* aged" OR "diabete* senior*" OR "elderly with diabetes" OR "elderly patient* with type 2 diabete*")) AND ALL=(diet* OR nutrition* OR "health diet*" OR activity OR exercise* OR lifestyle*)) AND ALL=("kidney* solution*" OR "chronic kidney*" OR kidney* OR "chronic kidney disease" OR CKD OR "diabetic kidney disease" OR DKD OR "kidney disease") \| 70 results; Cochrane \| (older NEXT adult* NEXT with NEXT diabete* OR senior* NEXT with NEXT diabete* OR geriatric* NEXT with NEXT diabete* OR diabete* NEXT old* OR diabete* NEXT elder* OR diabete* NEXT aged OR diabete* NEXT senior* OR elderly NEXT with NEXT diabetes OR elderly NEXT patient* NEXT with NEXT type 2 NEXT diabete*) AND (diet* OR nutrition* OR health NEXT diet* OR activity OR exercise* OR lifestyle*) AND (kidney* NEXT solution* OR chronic NEXT kidney* OR kidney* OR chronic NEXT kidney NEXT disease OR CKD OR diabetic NEXT kidney NEXT disease OR DKD OR kidney NEXT disease) \| 19 results | methodology, table 1 |
| Study risk of bias assessment | 11 | Two independent reviewers assessed methdological quality using MMAT. | methodology |
| Effect measures | 12 | Considering the methodological diversity of the included studies, study quality was appraised using the Mixed Methods Appraisal Tool (MMAT), 2018 version. The MMAT is specifically designed for systematic reviews that include multiple research designs and enables the assessment of qualitative, quantitative, and mixed-methods studies within a single framework. The included studies comprised randomized controlled trials, quantitative non-randomized studies, and quantitative descriptive studies. Accordingly, only the corresponding MMAT domains were applied. | methodology |
| Synthesis methods | 13a | Describe the processes used to decide which studies were eligible for each synthesis (e.g. tabulating the study intervention characteristics and comparing against the planned groups for each synthesis (item #5)). The quality of those total 31 articles was systematically assessed using the MMAT checklist, as presented in the table. The MMAT assessment evaluated methodological rigor, appropriateness of study design, sampling strategy, data collection procedures, outcome measurement, risk of bias, and interpretation of findings. Two reviewers independently conducted the quality assessment, and any disagreements were resolved through discussion to ensure consistency and reliability. Quality appraisal outcomes were not used as exclusion criteria; rather, they informed interpretive caution during the synthesis and discussion of findings.   | data sources and search strategy  , table 3 |
|  | 13b | Data conversions and coding. |  |
|  | 13c | Results from individual studies and syntheses were tabulated using summary tables that highlighted key outcomes, effect measures, and study characteristics. |  |
|  | 13d | Results were synthesized using a narrative synthesis approach, which was chosen due to the heterogeneity in study designs, populations, and outcomes. This method allowed for a detailed qualitative comparison of findings across studies, highlighting patterns, themes, and relationships within the data without relying on statistical aggregation. |  |
|  | 13e | N/A |  |
|  | 13f | N/A |  |
| Reporting bias assessment | 14 | N/A |  |
| Certainty assessment | 15 | Certainty in the body of evidence for each outcome was assessed using the CASP. |  |
| **RESULTS** | | |  |
| Study selection | 16a |   A total of 1,093 records were retrieved and exported to Microsoft Excel for duplicate removal. After removing 318 duplicates, 775 records remained for title screening. 635 unrelated articles were removed based on the title screen. The remaining 140 articles were screened through abstract review; 67 articles were removed in this process. The remaining 73 articles were thoroughly assessed by the two independent reviewers and only those articles meeting the criteria were included. In case of a contrast of opinions, other reviewers were consulted to resolve any disparity in the result by voting. However, any unresolved conflicts were ultimately addressed through consensus, ensuring that all decisions were mutually agreed upon by the research team. Secondary screening was performed by reading the full text of the articles thoroughly. Furthermore, back screening was done by checking the references for the potential articles. Finally, 31 selected studies were included in systematic review. | figure 1 |
|  | 16b | The remaining 31 articles were assessed for eligibility and extracted for literature review. Table presents in 13a describes the results of the CASP appraisal for all included articles. |  |
| Study characteristics | 17 | Cite each included study and present its characteristics.   | table 4-7 |
| Risk of bias in studies | 18 | To evaluate the overall certainty of the evidence across the included studies, the Grading of Recommendations Assessment, Development and Evaluation (GRADE) framework was applied. The results of the GRADE assessment are presented in Table 5 in accordance with established GRADE guidelines. Overall, the certainty of the evidence ranged from moderate to very low across the four thematic categories. The dietary patterns and diet quality theme was rated as having moderate-certainty evidence because it included relatively consistent findings from one long-term randomized controlled trial together with several well-conducted observational studies. In contrast, the remaining three themes were rated as low- or very low-certainty evidence because of methodological heterogeneity, the predominance of observational studies and small intervention trials, variations in intervention and exposure characteristics, and imprecision in the reported renal outcome measures. | methodology |
| Results of individual studies | 19 | Details, please refer to supporting document [1] For each theme the results of each study is listed below:  16. Podadera-Herreros A, Arenas-de Larriva AP, Gutierrez-Mariscal FM, Alcala-Diaz JF, Ojeda-Rodriguez A, Rodriguez-Cantalejo F, et al. Mediterranean diet as a strategy for preserving kidney function in patients with coronary heart disease with type 2 diabetes and obesity: a secondary analysis of CORDIOPREV randomized controlled trial. Nutr Diabetes. 2024;14(1):27. doi:10.1038/s41387-024-00285-3  17. Ding Z, Wu X, Liu C, Ying R, Zhang Y, Zhang S, et al. Associations between dietary patterns and renal impairment in individuals with diabetes: a cross-sectional study. J Hum Nutr Diet. 2024;37(1):193–202. doi:10.1111/jhn.13245  18. Cao L, Yu P, Zhang L, Yao Q, Zhou F, Li X, et al. Association between dietary patterns and chronic kidney disease in elderly patients with type 2 diabetes: a community-based cross-sectional study. Nutr J. 2025;24(1):1. doi:10.1186/s12937-024-01070-9  19. Meng X, Chen X, Zhang B, Wang J. Comparative analysis of dietary pattern indices and their associations with chronic kidney disease: a comprehensive analysis of NHANES data (2000–2020). Ren Fail. 2025;47(1):2540565. doi:10.1080/0886022X.2025.2540565  20. Wilkinson TJ, Lightfoot CJ, Smith AC. Comparison of dietary patterns and daily food intake across kidney disease stages in England: an a-posteriori cluster analysis. J Ren Nutr. 2025;35(1):90–102. doi:10.1053/j.jrn.2024.07.010  21. Rouhani MH, Mortazavi Najafabadi M, Moeinzadeh F, Esmaillzadeh A, Feizi A, Azadbakht L. Comparison of three diet quality indices for patients with chronic kidney disease. Arch Iran Med. 2017;20(8):474–480.  22. Tauchi E, Hanai K, Babazono T. Effects of dietary protein intake on renal outcome and mortality in patients with advanced diabetic nephropathy. Clin Exp Nephrol. 2020;24(2):119–125. doi:10.1007/s10157-019-01796-5  23. Malhotra R, Cavanaugh KL, Blot WJ, Ikizler TA, Lipworth L, Kabagambe EK. Higher protein intake is associated with increased risk for incident end-stage renal disease among blacks with diabetes in the Southern Community Cohort Study. Nutr Metab Cardiovasc Dis. 2016;26(12):1079–1087. doi:10.1016/j.numecd.2016.07.009  24. Møller G, Rikardt Andersen J, Ritz C, Silvestre MP, Navas-Carretero S, Jalo E, et al. Higher protein intake is not associated with decreased kidney function in pre-diabetic older adults following a one-year intervention: a preview sub-study. Nutrients. 2018;10(1):54. doi:10.3390/nu10010054  25. Piccoli GB, Ventrella F, Capizzi I, Vigotti FN, Mongilardi E, Grassi G, et al. Low-protein diets in diabetic chronic kidney disease (CKD) patients: are they feasible and worth the effort? Nutrients. 2016;8(10):649. doi:10.3390/nu8100649  26. Bellizzi V, Calella P, Hernández JN, González VF, Lira SM, Torraca S, et al. Safety and effectiveness of low-protein diet supplemented with ketoacids in diabetic patients with chronic kidney disease. BMC Nephrol. 2018;19(1):110. doi:10.1186/s12882-018-0914-5  27. Saran R, Padilla RL, Gillespie BW, Heung M, Hummel SL, Derebail VK, et al. A randomized crossover trial of dietary sodium restriction in stage 3–4 CKD. Clin J Am Soc Nephrol. 2017;12(3):399–407. doi:10.2215/CJN.01120216  28. Kanauchi N, Ookawara S, Ito K, Mogi S, Yoshida I, Kakei M, et al. Factors affecting the progression of renal dysfunction and the importance of salt restriction in patients with type 2 diabetic kidney disease. Clin Exp Nephrol. 2015;19(6):1120–1126. doi:10.1007/s10157-015-1118-y  29. Zainordin NA, Eddy Warman NA, Mohamad AF, Abu Yazid FA, Ismail NH, Chen XW, et al. Safety and efficacy of very low carbohydrate diet in patients with diabetic kidney disease: a randomized controlled trial. PLoS One. 2021;16(10):e0258507. doi:10.1371/journal.pone.0258507  30. Zainordin NA, Warman NAE, Mohamad AF, Yazid FAA, Ismail NH, Wee CX, et al. The effects very low carbohydrate diet (VLCBD) on renal outcomes in diabetic kidney disease patients: a 12-week randomized controlled trial. J ASEAN Fed Endocr Soc. 2021;36:62. doi:10.15605/jafes.036.S93  31. Zainordin NA, Warman NAE, Mohamad AF, Yazid FAA, Ismail NH, Wee CX, et al. The very low carbohydrate diet (VLCBD): short-term metabolic efficacy in diabetic kidney disease patients. J ASEAN Fed Endocr Soc. 2021;36:7. doi:10.15605/jafes.036.S7  32. Tay J, Thompson CH, Luscombe-Marsh ND, Noakes M, Buckley JD, Wittert GA, et al. Long-term effects of a very low carbohydrate compared with a high carbohydrate diet on renal function in individuals with type 2 diabetes: a randomized trial. Medicine (Baltimore). 2015;94(47):e2181. doi:10.1097/MD.0000000000002181  33. Khatami PG, Soleimani A, Sharifi N, Aghadavod E, Asemi Z. The effects of high-dose vitamin E supplementation on biomarkers of kidney injury, inflammation, and oxidative stress in patients with diabetic nephropathy: a randomized, double-blind, placebo-controlled trial. J Clin Lipidol. 2016;10(4):922–929. doi:10.1016/j.jacl.2016.02.021  34. Jiménez-Osorio AS, García-Niño WR, González-Reyes S, Álvarez-Mejía AE, Guerra-León S, Salazar-Segovia J, et al. The effect of dietary supplementation with curcumin on redox status and Nrf2 activation in patients with nondiabetic or diabetic proteinuric chronic kidney disease: a pilot study. J Ren Nutr. 2016;26(4):237–244. doi:10.1053/j.jrn.2016.01.013  35. Miraghajani M, Zaghian N, Dehkohneh A, Mirlohi M, Ghiasvand R. Probiotic soy milk consumption and renal function among type 2 diabetic patients with nephropathy: a randomized controlled clinical trial. Probiotics Antimicrob Proteins. 2019;11(1):124–132. doi:10.1007/s12602-017-9325-3  36. Rouhani MH, Najafabadi MM, Surkan PJ, Esmaillzadeh A, Feizi A, Azadbakht L. Dietary inflammatory index and its association with renal function and progression of chronic kidney disease. Clin Nutr ESPEN. 2019;29:237–241. doi:10.1016/j.clnesp.2018.09.001  37. Leehey DJ, Collins E, Kramer HJ, Cooper C, Butler J, McBurney C, et al. Structured exercise in obese diabetic patients with chronic kidney disease: a randomized controlled trial. Am J Nephrol. 2016;44(1):54–62. doi:10.1159/000447703  38. Nataraj M, Maiya GA, Nagaraju SP, Shastry BA, Shivashankara KN, Shetty S, et al. Effect of exercise-based rehabilitation on functional capacity and renal function in type 2 diabetes mellitus with nephropathy: a randomized controlled trial. Int Urol Nephrol. 2024;56(8):2671–2682. doi:10.1007/s11255-024-03987-w  39. Kimura T, Washida N, Ohtsuki S, Sugita K, Hosoya K, Uchiyama K. A multi-center randomized controlled trial to investigate potential effects of exercise therapy on renal function stratified by renal disorders and renal pathology: beneficial or harmful effect in immunoglobulin A nephropathy. Clin Exp Nephrol. 2024;28(6):539–546. doi:10.1007/s10157-024-02461-2  40. Aydemir N, Pike MM, Alsouqi A, Headley SAE, Tuttle K, Evans EE, et al. Effects of diet and exercise on adipocytokine levels in patients with moderate to severe chronic kidney disease. Nutr Metab Cardiovasc Dis. 2020;30(8):1375–1381. doi:10.1016/j.numecd.2020.04.012  41. Beetham KS, Howden EJ, Isbel NM, Coombes JS. Agreement between cystatin-C and creatinine based eGFR estimates after a 12-month exercise intervention in patients with chronic kidney disease. BMC Nephrol. 2018;19(1):366. doi:10.1186/s12882-018-1146-4  42. Anderton N, Giri A, Wei G, Marcus RL, Chen X, Bjordahl T, et al. Sedentary behavior in individuals with diabetic chronic kidney disease and maintenance hemodialysis. J Ren Nutr. 2015;25(4):364–370. doi:10.1053/j.jrn.2015.01.018  43. Palakodeti S, Uratsu CS, Schmittdiel JA, Grant RW. Changes in physical activity among adults with diabetes: a longitudinal cohort study of inactive patients with type 2 diabetes who become physically active. Diabet Med. 2015;32(8):1051–1057. doi:10.1111/dme.12748  44. Wong KK, Velasquez A, Powe NR, Tuot DS. Association between health literacy and self-care behaviors among patients with chronic kidney disease. BMC Nephrol. 2018;19(1):196. doi:10.1186/s12882-018-0988-0  45. Shah B, Kirpalani A, Sunder S, Gupta A, Khanna U, Chafekar D, et al. A prospective, multi-centre, observational study to examine kidney disease progression in adults with chronic kidney disease—CKDOD: study design and preliminary results. BMC Nephrol. 2015;16:215. doi:10.1186/s12882-015-0191-5 | supporting document [1] |
| Results of syntheses | 20a | This systematic review provides strong evidence that healthy dietary practices and structured physical activity significantly improve key clinical outcomes in elderly patients with Type II diabetes and chronic kidney disease. Improvements were observed in eGFR, HbA1c, blood pressure, and physical function, indicating a meaningful impact on both renal and metabolic health.  These findings reinforce the critical role of non-pharmacological interventions in chronic disease management. However, future research should aim to identify the most effective intervention components, evaluate long-term sustainability, and ensure broader applicability across diverse populations and healthcare settings. Integrating evidence-based lifestyle programs into standard CKD care pathways could substantially improve quality of life and delay disease progression in this vulnerable population. | results |
|  | 20b | N/A |  |
|  | 20c | N/A |  |
|  | 20d | N/A |  |
| Reporting biases | 21 | N/A |  |
| Certainty of evidence | 22 | The remaining 31 articles were assessed for eligibility and extracted for literature review. |  |
| **DISCUSSION** | | |  |
| Discussion | 23a | This systematic review found that healthy diet and physical activity interventions produced significant benefits in managing chronic kidney disease (CKD) among elderly individuals with Type II diabetes. Improvements were observed in eGFR, systolic blood pressure, glycemic control, and body weight, suggesting that lifestyle-based interventions can slow CKD progression and improve overall metabolic health. | results |
|  | 23b | The evidence included in the review was limited by literature review, and heterogeneity in study designs. | results |
|  | 23c | several limitations warrant discussion. First, the heterogeneity of intervention components and outcome measurements introduces variability. Second, some included studies were at high risk of bias, particularly in reporting and attrition domains. Lastly, the generalizability of findings may be limited due to geographic concentration of trials in high-income countries. | results |
|  | 23d | These findings reinforce the critical role of non-pharmacological interventions in chronic disease management. However, future research should aim to identify the most effective intervention components, evaluate long-term sustainability, and ensure broader applicability across diverse populations and healthcare settings. Integrating evidence-based lifestyle programs into standard CKD care pathways could substantially improve quality of life and delay disease progression in this vulnerable population. | results |
| **OTHER INFORMATION** | | |  |
| Registration and protocol | 24a | The review was registered. (registered ID: CRD420251246555) |  |
|  | 24b | That a protocol was not prepared. |  |
|  | 24c | No amendments to information provided at registration or in the protocol. |  |
| Support | 25 | This research received no external funding. |  |
| Competing interests | 26 | No potential conflict of interest was reported by the author(s). |  |
| Availability of data, code and other materials | 27 | The data that support the findings of this study are available from the corresponding author, upon reasonable request. Meanwhile, all relevant data are within the manuscript and its Supporting Information files. Data documents are available in Figshare at: https://figshare.com/s/0bb249b2683a9cb0a484 | Supplementary information |

*From:*  Page MJ, McKenzie JE, Bossuyt PM, Boutron I, Hoffmann TC, Mulrow CD, et al. The PRISMA 2020 statement: an updated guideline for reporting systematic reviews. BMJ 2021;372:n71. doi: 10.1136/bmj.n71
