# Supplementary material for: Dietary patterns and physical activity in relation to renal and metabolic outcomes among older adults with type 2 diabetes and chronic kidney disease: a systematic review
Source: Front Nutr. 2026 Sep 16;13:1858619. doi: 10.3389/fnut.2026.1858619 (PMC13623551; doi:10.3389/fnut.2026.1858619)
Supplement: Supplementary file 2 [file Table_2.DOCX]

| **Title** | **Dietary Patterns and Physical Activity in Relation to Renal and Metabolic Outcomes among Older Adults with Type 2 Diabetes and Chronic Kidney Disease: A Systematic Review** | | |
| --- | --- | --- | --- |
| **Main keyword in PIO** | **Term** | **Source of retrieval** | **Selected keywords** |
| P | Older adults with type 2 diabetes and chronic kidney disease. | Previous systematic reviews | ("older adult* with diabete*" OR "senior* with diabete*" OR "geriatric* with diabete*" OR "diabete* old*" OR "diabete* elder*" OR "diabete* aged" OR "diabete* senior*" OR "elderly with diabetes" OR "elderly patient* with type 2 diabete*") |
| I | Diet and lifestyle-related factors, including dietary practices, nutrition, healthy diet, physical activity, exercise, and lifestyle interventions. | Previous systematic reviews  Market Research  Consultation (experienced designers) | (diet* OR nutrition* OR "health diet*" OR activity OR exercise* OR lifestyle*) |
| ~~C~~ | Not applied | Not applied | Not applied |
| O | Kidney-related outcomes, including chronic kidney disease, diabetic kidney disease, kidney disease, and other renal health outcomes associated with diet, nutrition, physical activity, or lifestyle factors. | MeSH term | ("kidney* solution*" OR "chronic kidney*" OR kidney* OR "chronic kidney disease" OR CKD OR "diabetic kidney disease" OR DKD OR "kidney disease") |

**Supplementary Material 2 PICO**

**PICO Question:** Among older adults with Type 2 diabetes and chronic kidney disease (Population), how are dietary interventions, dietary patterns, physical activity interventions, and lifestyle-related exposures (Intervention/Exposure) associated with renal and metabolic outcomes (Outcomes)?

**Background:** Lifestyle modification is increasingly recognized as a critical component in the management of chronic kidney disease (CKD), particularly among elderly individuals with Type 2 diabetes, a population at high risk of disease progression and comorbidities. While pharmacological treatment remains central, growing evidence highlights the role of dietary patterns and physical activity in improving renal and metabolic outcomes. However, the extent and consistency of these effects remain unclear, particularly in older diabetic populations.

**Population:** Older adults with Type 2 diabetes mellitus (T2DM) and chronic kidney disease (CKD), including studies involving broader CKD or T2DM populations that provided clinically relevant indirect evidence applicable to elderly individuals with diabetic CKD.

**Problem:** Older adults with Type 2 diabetes are at high risk of CKD progression, declining renal function, poor glycaemic control, hypertension, and cardiovascular complications. Although dietary modification and physical activity are widely recommended as non-pharmacological management strategies, existing evidence is heterogeneous, and the effectiveness of different dietary patterns, nutritional strategies, and physical activity interventions on renal and metabolic outcomes in this population remains uncertain.

**Outcomes:** Renal outcomes (e.g., estimated glomerular filtration rate [eGFR], albuminuria/proteinuria, CKD progression, renal function) and metabolic outcomes (e.g., HbA1c, blood pressure, body weight, insulin sensitivity, inflammatory biomarkers, physical function, and quality of life).

| **TITLE-ABS-KEY** | **Scopus** | TITLE-ABS-KEY ( ( "older adult* with diabete*" OR "senior* with diabete*" OR "geriatric* with diabete*" OR "diabete* old*" OR "diabete* elder*" OR "diabete* aged" OR "diabete* senior*" OR "elderly with diabetes" OR "elderly patient* with type 2 diabete*" ) AND ( diet* OR nutrition* OR "health diet*" OR activity OR exercise* OR lifestyle* ) AND ( "kidney* solution*" OR "chronic kidney*" OR kidney* OR "chronic kidney disease" OR CKD OR "diabetic kidney disease" OR DKD OR "kidney disease" ) ) |
| --- | --- | --- |
|  | **Web of Science** | ((ALL=("older adult* with diabete*" OR "senior* with diabete*" OR "geriatric* with diabete*" OR "diabete* old*" OR "diabete* elder*" OR "diabete* aged" OR "diabete* senior*" OR "elderly with diabetes" OR "elderly patient* with type 2 diabete*")) AND ALL=(diet* OR nutrition* OR "health diet*" OR activity OR exercise* OR lifestyle*)) AND ALL=("kidney* solution*" OR "chronic kidney*" OR kidney* OR "chronic kidney disease" OR CKD OR "diabetic kidney disease" OR DKD OR "kidney disease") |
|  | **PubMed** | ("older adult* with diabete*" OR "senior* with diabete*" OR "geriatric* with diabete*" OR "diabete* old*" OR "diabete* elder*" OR "diabete* aged" OR "diabete* senior*" OR "elderly with diabetes" OR "elderly patient* with type 2 diabete*") AND (diet* OR nutrition* OR "health diet*" OR activity OR exercise* OR lifestyle*) AND ("kidney* solution*" OR "chronic kidney*" OR kidney* OR "chronic kidney disease" OR CKD OR "diabetic kidney disease" OR DKD OR "kidney disease") |
|  | **EMBASE** | ('older adult* with diabete*' OR 'senior* with diabete*' OR 'geriatric* with diabete*' OR 'diabete* old*' OR 'diabete* elder*' OR 'diabete* aged' OR 'diabete* senior*' OR 'elderly with diabetes' OR 'elderly patient* with type 2 diabete*') AND (diet* OR nutrition* OR 'health diet*' OR 'activity'/exp OR activity OR exercise* OR lifestyle*) AND ('kidney* solution*' OR 'chronic kidney*' OR kidney* OR 'chronic kidney disease'/exp OR 'chronic kidney disease' OR ckd OR 'diabetic kidney disease'/exp OR 'diabetic kidney disease' OR dkd OR 'kidney disease'/exp OR 'kidney disease') |
|  | **Cochrane** | (older NEXT adult* NEXT with NEXT diabete* OR senior* NEXT with NEXT diabete* OR geriatric* NEXT with NEXT diabete* OR diabete* NEXT old* OR diabete* NEXT elder* OR diabete* NEXT aged OR diabete* NEXT senior* OR elderly NEXT with NEXT diabetes OR elderly NEXT patient* NEXT with NEXT type 2 NEXT diabete*) AND (diet* OR nutrition* OR health NEXT diet* OR activity OR exercise* OR lifestyle*) AND (kidney* NEXT solution* OR chronic NEXT kidney* OR kidney* OR chronic NEXT kidney NEXT disease OR CKD OR diabetic NEXT kidney NEXT disease OR DKD OR kidney NEXT disease) |

| **Database** | **Results (2015 Jan-2025 Dec)** |
| --- | --- |
| **PubMed** | 918 results |
| **EMBASE** | 11 results |
| **Scopus** | 75 results |
| **Web of Science** | 70 results |
| **Cochrane** | 19 results |
